# Mechanisms of the host immune response and helminth-induced pathology during *Trichobilharzia regenti* (Schistosomatidae) neuroinvasion in mice

**DOI:** 10.1371/journal.ppat.1010302

**Published:** 2022-02-04

**Authors:** Tomáš Macháček, Roman Leontovyč, Barbora Šmídová, Martin Majer, Oldřich Vondráček, Iveta Vojtěchová, Tomáš Petrásek, Petr Horák

**Affiliations:** 1 Department of Parasitology, Faculty of Science, Charles University, Prague, Czechia; 2 National Institute of Mental Health, Klecany, Czechia; 3 Laboratory of Neurophysiology of Memory, Institute of Physiology of the Czech Academy of Sciences, Prague, Czechia; National Institutes of Health Clinical Center, UNITED STATES

## Abstract

Helminth neuroinfections represent serious medical conditions, but the diversity of the host-parasite interplay within the nervous tissue often remains poorly understood, partially due to the lack of laboratory models. Here, we investigated the neuroinvasion of the mouse spinal cord by *Trichobilharzia regenti* (Schistosomatidae). Active migration of *T*. *regenti* schistosomula through the mouse spinal cord induced motor deficits in hindlimbs but did not affect the general locomotion or working memory. Histological examination of the infected spinal cord revealed eosinophilic meningomyelitis with eosinophil-rich infiltrates entrapping the schistosomula. Flow cytometry and transcriptomic analysis of the spinal cord confirmed massive activation of the host immune response. Of note, we recorded striking upregulation of the major histocompatibility complex II pathway and M2-associated markers, such as arginase or chitinase-like 3. Arginase also dominated the proteins found in the microdissected tissue from the close vicinity of the migrating schistosomula, which unselectively fed on the host nervous tissue. Next, we evaluated the pathological sequelae of *T*. *regenti* neuroinvasion. While no demyelination or blood-brain barrier alterations were noticed, our transcriptomic data revealed a remarkable disruption of neurophysiological functions not yet recorded in helminth neuroinfections. We also detected DNA fragmentation at the host-schistosomulum interface, but schistosomula antigens did not affect the viability of neurons and glial cells *in vitro*. Collectively, altered locomotion, significant disruption of neurophysiological functions, and strong M2 polarization were the most prominent features of *T*. *regenti* neuroinvasion, making it a promising candidate for further neuroinfection research. Indeed, understanding the diversity of pathogen-related neuroinflammatory processes is a prerequisite for developing better protective measures, treatment strategies, and diagnostic tools.

## Author summary

Infections of the human central nervous system by parasitic worms, such as pork tapeworm or rat lungworm, are a health threat, especially in communities with poor hygiene and sanitation. Understanding the diversity of host-parasite interactions, possibly leading to development of effective treatment or vaccine, requires suitable animal models. Our study deeply characterized the neuroinvasion of the mouse spinal cord by the neuropathogenic flatworm *Trichobilharzia regenti*. Its behavior within the nervous tissue (active migration, eager feeding) and clinical outcome of the infection (weight loss, motor dysfunctions, strong neuroinflammation) resemble those observed in human diseases. Using an integrative approach, we elucidated how the mice fight against the parasites and which mechanisms could be responsible for the pathological sequelae. Moreover, we identified pathways potentially involved in the reparation of the injured nervous tissue. All these features make *T*. *regenti* in mice a promising candidate for further research in parasitology, and neuroimmunology.

## Introduction

Parasitic helminths often invade the central nervous system (CNS) of mammals, including humans. Invasion of the CNS is either a natural part of their somatic migration or represents an unwanted, ectopic localization [1]. The clinical manifestation of helminth neuroinfections ranges from mostly asymptomatic to very severe, leading to sensory or cognitive deficits and seizures or epilepsy [2–4]. Many factors, such as parasite burden or localization within the CNS, influence the course and outcome of the neuroinfection [5]. Moreover, the host immune response affects helminth growth and survival but might also participate in the pathogenesis or behavioral alterations [6,7]. Therefore, a deep understanding of host-parasite immune interactions is required to develop better protective measures, treatment strategies, and diagnostic tools.

The availability of suitable laboratory models dictates the primary orientation of the helminth neuroinfection research. Neurocysticercosis, cerebral angiostrongylosis, and neurotoxocarosis are hence in focus as their models have been established [8–13]. On the contrary, a valid model representing, e.g., the neurological form of schistosomosis or echinococcosis is lacking [14–16]. Novel model species for studying helminth neuroinfections are thus needed to reveal the diversity of host-parasite interactions and (immuno-)pathological sequelae. Even if they do not fully mirror any specific human disease, investigating their interactions with the host could bring valuable insights. This is also the case of some filarial or intestinal model helminths that only partially reflect the human diseases but are useful after integrating the experimental findings [17,18]. Regarding the neurotropic helminths, the species naturally invading the CNS should be considered as no artificial manipulations, such as injections of parasites into the CNS [12,16], are required.

*Trichobilharzia regenti* is a schistosome that naturally migrates through the CNS of birds and mammals (birds are the definitive hosts, whereas mammals represent the accidental hosts). After penetration of the host skin, the newly transformed schistosomula find the peripheral nerves *via* which they enter the spinal cord [19,20]. Here they eagerly feed on the nervous tissue, but no significant demyelination is observed [21–23]. However, leg paralysis is often reported in ducks and immunocompromised mice due to a higher schistosomula burden and/or their prolonged persistence within the CNS [21,22]. On the contrary, immunocompetent mice effectively control *T*. *regenti* neuroinvasion. Indeed, recruited peripheral leukocytes entrap and destroy the schistosomula in the spinal cord 7–14 days post infection (dpi) [22,24]. The CNS-resident microglia are also activated [22] but do not seem to harm the living schistosomula, e.g., by the production of nitric oxide [25,26]. Even though living schistosomula are rarely found after 21 dpi [22,24], the host effector immune mechanisms remain unclear.

**Fig 1:**
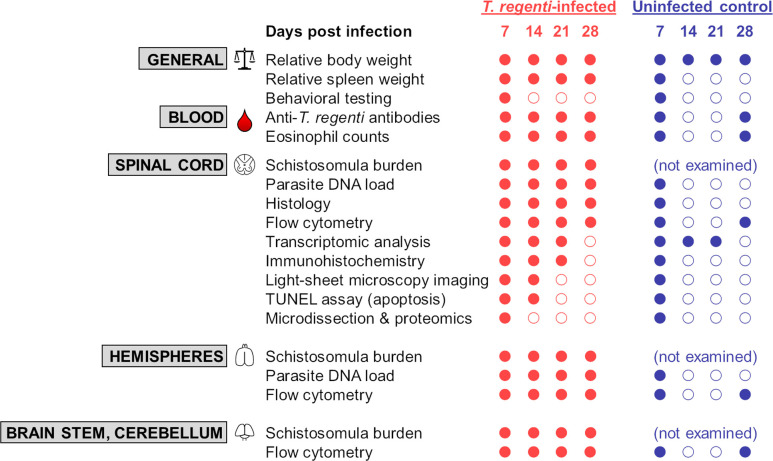
Summary of experimental procedures. C57BL/6J female mice were infected with *T*. *regenti* cercariae and examined at desired time points (indicated by filled red dots). Uninfected mice were used as controls; particular age-matching is shown for each analysis (filled blue dots). Empty dot means that the analysis was not performed at the respective time point.

Here we employed a complex approach, ranging from behavioral testing to ‘omics’ analyses (Fig 1), to explore mechanisms of the host immune response and helminth-induced pathology in the CNS of mice infected with *T*. *regenti*. Our comprehensive insight allows us to compare *T*. *regenti*- neuroinvasion with other medically important helminth neuroinfections and uncover the diversity of host-parasite interactions within the nervous tissue. In this regard, the remarkable disruption of neurophysiological functions (not observed in other helminths yet) and a strong M2 polarization in *T*. *regenti*-infected spinal cords should be of particular interest.

## Results

### *T*. *regenti* triggered peripheral immunity and invaded the CNS, especially the spinal cord

**Fig 2:**
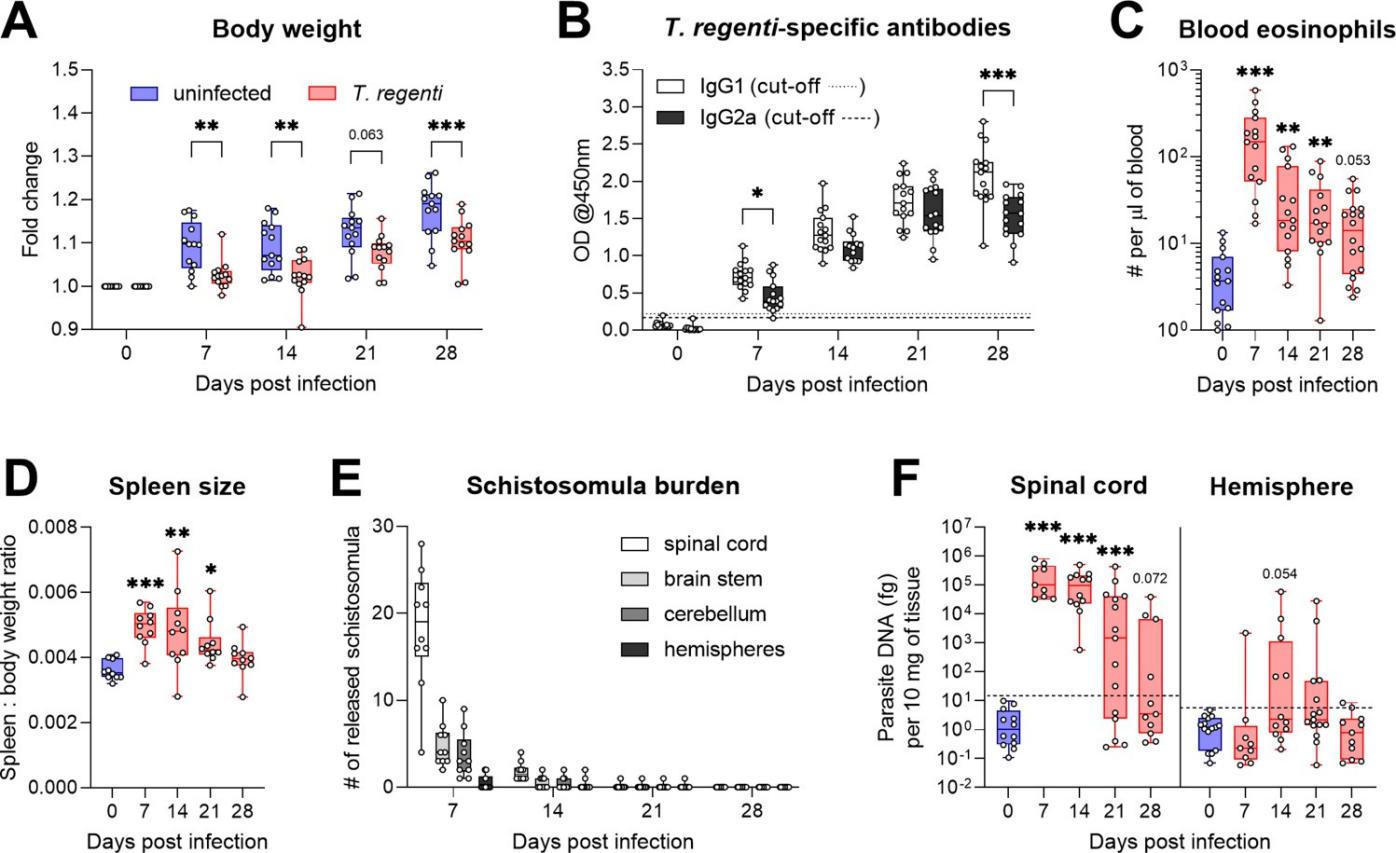
General features of *T*. *regenti* infection in C57BL/6J mice. **(A)** Infected mice exhibited a lower gain in body weight than age-matched uninfected controls. **(B)** The levels of *T*. *regenti*-specific IgG1 and IgG2a detected in mouse sera rose steadily throughout the infection. Th2-associated IgG1 dominated over Th1-associated IgG2a, especially 28 dpi. **(C)** Blood eosinophil counts have increased in infected mice 7–21 dpi. **(D)** Spleen enlargement was observed in infected mice 7–21 dpi. **(E)** Within the CNS, most viable schistosomula were found 7 dpi being localized predominantly in the spinal cord. The amount of released schistosomula remarkably decreased at later time points. **(F)** Contrary to those from hemispheres, *T*. *regenti* DNA was detected in most of the spinal cord tissue samples 7–21 dpi. In each graph, points show data from individual mice. Pooled data from 2–3 independent experiments are shown. Data were evaluated by 2-way ANOVA for repeated measures and Šidák’s test (A), ordinary 2-way ANOVA and Šidák’s test (B, F), Kruskal-Wallis and Dunn’s test (C), or ordinary 1-way ANOVA and Dunnett’s test (D); *p<0.05, **p<0.01, ***p<0.001.

First, we briefly characterized general features of *T*. *regenti* infection to establish the model and validate its reproducibility in C57BL/6J mice 7, 14, 21, and 28 dpi. The infection did not alter mouse survival, but the infected individuals exhibited a decreased gain in body weight (Fig 2A; p(7 dpi) = 0.0022; p(14 dpi) = 0.0024; p(28 dpi) = 0.0009) despite having the same feed consumption rate as the uninfected group. The infected mice produced both parasite-specific IgG1 and IgG2a as soon as 7 dpi. Levels of Th2-associated IgG1 rose continuously and prevailed over Th1-associated IgG2a (Fig 2B), especially 28 dpi (p < 0.0001). These observations of antibody response corroborate with the previous studies [27–29], which demonstrates reproducibility of the host immune reaction. The number of blood eosinophils peaked 7 dpi and remained elevated 14 and 21 dpi (Fig 2C; p(7 dpi) < 0.0001; p(14 dpi) = 0.0017; p(21 dpi) = 0.0053). A similar trend was observed in the case of spleen enlargement (Fig 2D; p(7 dpi) = 0.0007; p(14 dpi) = 0.0019; p(21 dpi) = 0.0456), which correlates with already reported increased numbers of splenic CD4+ and CD8+ T cells 7 and 14 dpi [29].

Due to *T*. *regenti* neurotropism, we further examined the distribution of schistosomula within the CNS, which has not been thoroughly done in C57BL/6J mice. The highest number of viable schistosomula was extracted 7 dpi (27.3 ± 9.8 per mouse, n = 10). They predominantly invaded the spinal cord (Fig 2E) but were also localized in the brain stem or the cerebellum. However, only 3 out of 10 mice harbored 1–2 schistosomula in the hemispheres. Markedly fewer schistosomula were released from the entire CNS 14 dpi (3.0 ± 2.1 per mouse, n = 10) and 21 dpi (4 schistosomula isolated from 2 mice out of 10); no schistosomula were recorded 28 dpi. Such migration pattern agrees with the historical records (obtained from other mouse strains) showing accumulation of schistosomula in the spinal cord and the highest burden within the CNS around 7 dpi [20,21]. To complement the data on schistosomula distribution, we also analyzed *T*. *regenti* DNA content in the spinal cord and hemisphere tissue samples. The parasite DNA was present in the spinal cords of all mice 7 and 14 dpi, and most of the mice 21 dpi (Fig 2F; p(7, 14, and 21 dpi) < 0.0001). On the contrary, the hemispheres seldom contained the parasite DNA; less than half of the samples were positive 14 and 21 dpi.

Collectively, our data demonstrated early activation of the peripheral immune response against *T*. *regenti*, which successfully invaded the CNS, especially the spinal cord, by 7 dpi.

### Motor function deficits affected the lower body of *T*. *regenti*-infected mice

**Fig 3:**
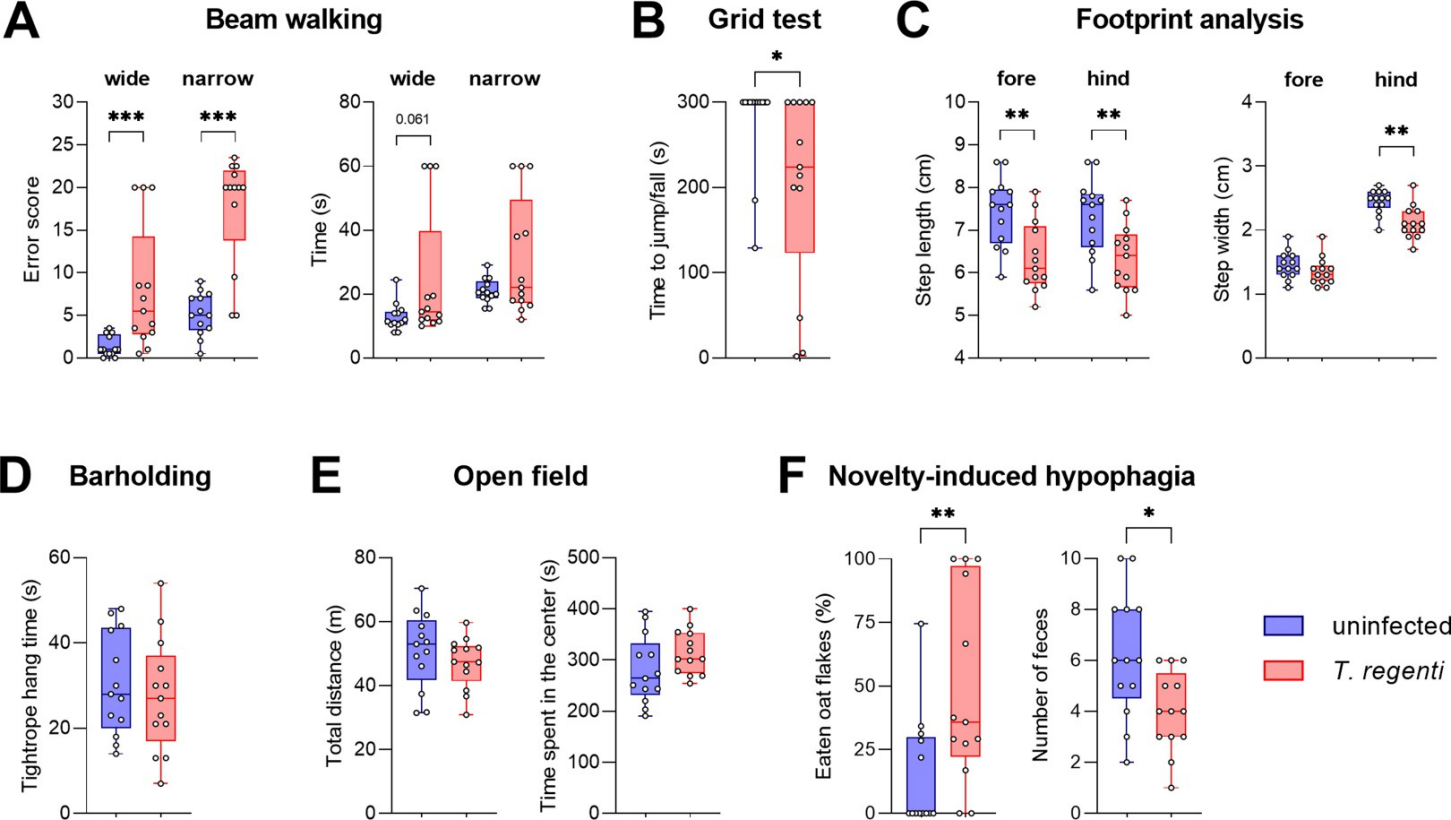
Effects of *T*. *regenti* infection on mouse behavior 7 dpi. **(A**) Beam walking on wide and narrow beams, impaired motor coordination in infected mice. **(B)** Grid test, worse endurance, and strength of the forelimbs and hindlimbs of infected mice. **(C)** Footprint analysis of forelimbs and hindlimbs, altered posture and gait in infected mice. **(D)** Bar holding, normal endurance, and strength of the forelimbs in both groups. **(E)** Open field, unaffected locomotor activity, and anxiety in both groups. **(F)** Novelty-induced hypophagia, higher amount of eaten oat flakes caused by stronger appetitive motivation, rather than increased anxiety of infected mice. In each graph, points show data from individual mice. Pooled data from 3 independent experiments are presented. Data were evaluated by Mann-Whitney test (A, B, F—eating) or unpaired t-test (C, D, E, F—feces); *p<0.05, **p<0.01, ***p<0.001.

The highest schistosomula burden was detected in the spinal cord 7 dpi. At this moment, we assessed the impact of neuroinvasion on various aspects of mouse behavior. Most importantly, the infected mice showed a deficit in motor functions. They made more errors when traversing a wide (p = 0.0006) or narrow (p < 0.0001) beam (Fig 3A and S1 Video), exhibited lower endurance when hanging upside-down on a grid (Fig 3B; p = 0.0313), and made shorter steps (Fig 3C; p(forelimbs) = 0.0027; p(hindlimbs) = 0.0059). Visual observations also showed altered tail posture in the infected mice (tail dragging, reduced tail motility). We suggest that motor functions of the lower body were affected as the performance in the bar holding task, depending predominantly on forelimb strength, was unaltered (Fig 3D), and step width was decreased in hindlimbs only (Fig 3C; p(forelimbs) = 0.2545; p(hindlimbs) = 0.001). However, general locomotor activity in the open field (Fig 3E), plus maze or Y-maze was normal, as well as spatial working memory in the Y-maze (S1 Fig). In the test of novelty-induced hypophagia, the infected mice ate more (p = 0.0094) and defecated less (p = 0.0117) than controls (Fig 3F). This behavior is more likely related to a stronger appetitive motivation than altered emotionality as we detected no signs of elevated anxiety (Fig 3E) or depression-like behavior in several other tests (S1 Fig). Collectively, our experiments showed that deficits in motor functions were the most evident behavioral sequelae of *T*. *regenti* neuroinvasion.

### Eosinophil-rich infiltrates entrapped and eliminated schistosomula in the spinal cord

**Fig 4:**
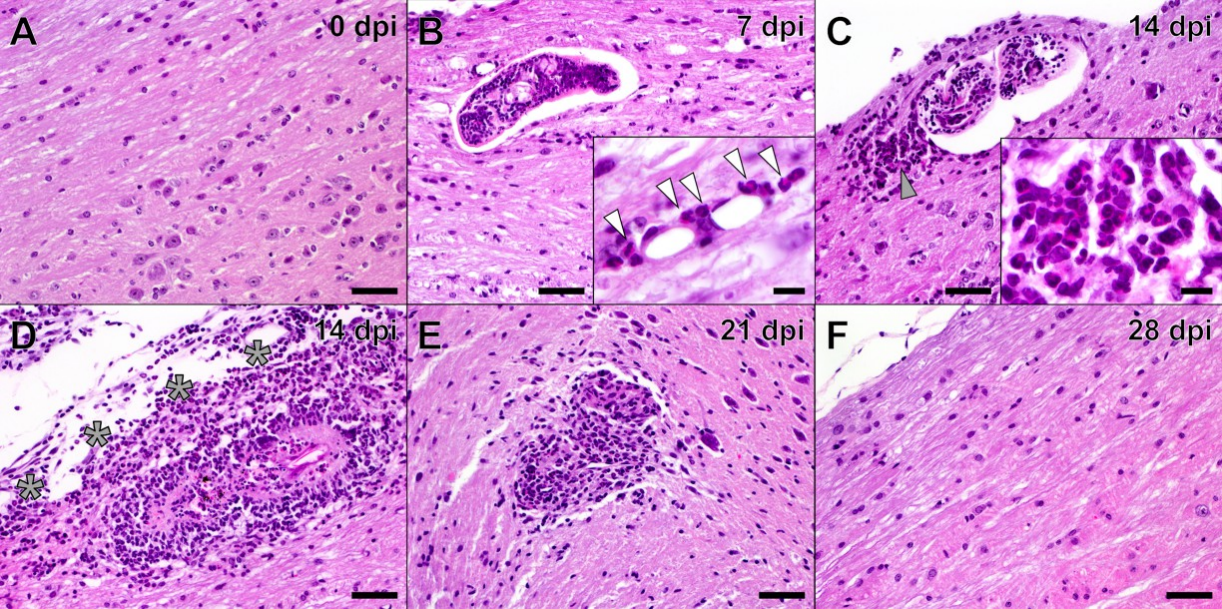
Histological examination of the *T*. *regenti*-infected spinal cord. **(A)** In the uninfected spinal cord, no leukocyte infiltration was apparent. **(B)** The intact schistosomulum in the white matter 7 dpi. No inflammatory cells surrounded the schistosomulum, but eosinophil extravasation was recorded in the adjacent areas (inset, eosinophils marked by white arrowheads). **(C)** The “rocket-tail” leukocyte cluster behind the schistosomulum 14 dpi. Eosinophils and mononuclear cells prevailed in the cluster (gray arrowhead and inset). **(D)** The inflammatory lesion around the destroyed schistosomulum 14 dpi. The adjoining leptomeningeal spaces (gray asterisks) were thickened and heavily inflamed. **(E)** The schistosomulum remnants within the fading inflammatory lesion 21 dpi. **(F)** The spinal cord tissue 28 dpi, no schistosomula residues or inflammatory foci were evident. Representative images from 2 independent experiments (each with 2–3 mice) per time point are shown. Scale bar (large image) = 50 μm, scale bar (inset) = 10 μm.

After assessing the general impact of *T*. *regenti* infection on mice, we performed a histological examination of the spinal cord to elucidate the course of the infection in the most affected segment of the CNS. Only intact schistosomula were observed 7 dpi being localized predominantly in the white matter (Fig 4B). Flattened cells with squeezed nuclei surrounded some of the schistosomula, while some were followed by “rocket-tail” leukocyte clusters (see below). Extravasation of leukocytes, mainly eosinophils (Fig 4B, inset), was recorded in the areas close to migrating schistosomula. The neuroinflammation culminated 14 dpi when no schistosomula were left unnoticed by the host immune cells. Of note, striking “rocket-tail” leukocyte clusters, consisting mainly of eosinophils and mononuclear cells (Fig 4C, inset), formed behind schistosomula (Fig 4C). Some parasites were still compact but had a thinner body wall and vacuolized internal tissues with condensed nuclei. However, some schistosomula were already damaged, and eosinophils, neutrophils, monocytes, and lymphocytes extensively infiltrated the lesions (Fig 4D). The adjacent leptomeningeal spaces were also thickened and heavily inflamed. The inflammatory foci around hardly distinguishable schistosomula or their remnants were still apparent 21 dpi (Fig 4E), but the leukocyte infiltration was fading away. Neither schistosomula residues nor inflammatory foci were detected 28 dpi (Fig 4F). Taken together, *T*. *regenti* neuroinvasion caused eosinophilic meningomyelitis with a peak 14 dpi. The cellular infiltrate was presumably essential for parasite elimination.

### Eosinophils were the most numerous cells infiltrating the entire CNS during *T*. *regenti* infection

**Fig 5:**
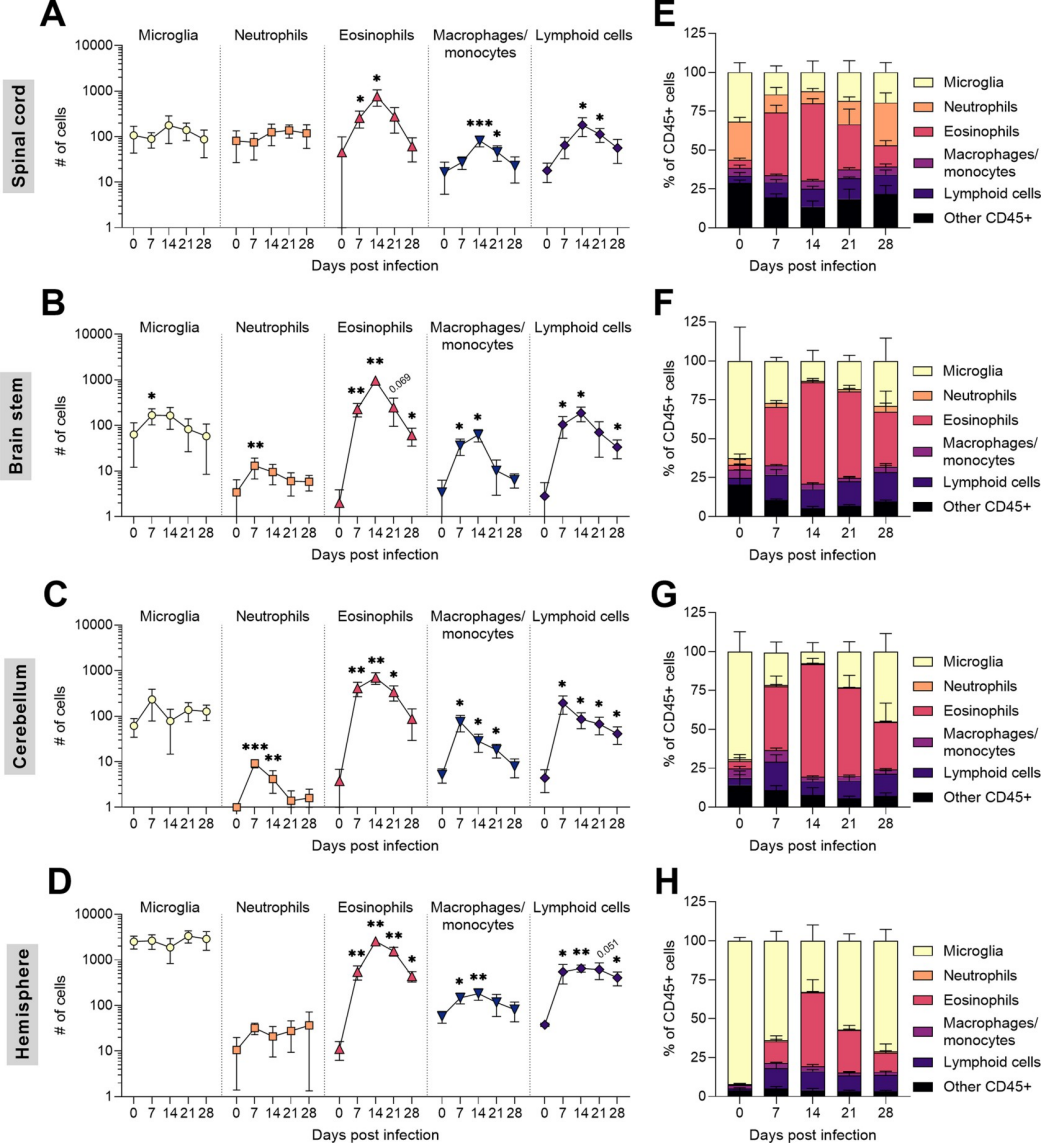
Dynamics of major immune cell populations in the mouse CNS during *T*. *regenti* infection. **(A–D)** Microglia, neutrophils, eosinophils, macrophages/monocyte, and lymphoid cells were analyzed by flow cytometry in the spinal cord (A), the brain stem (B), the cerebellum (C), and the hemisphere (D). In all examined segments, the most prominent increase was observed in eosinophil counts 14 dpi. Gating strategies are shown in S2 Text. Data (n = 5 per each time point) were evaluated by ordinary 1-way or Welch’s ANOVA followed by Dunnett’s test; *p<0.05, **p<0.01, ***p<0.001. **(E–H)** The relative proportion of the examined immune cell populations in the spinal cord (E), the brain stem (F), the cerebellum (G), and the hemisphere (H). Massive infiltration of peripheral leukocytes caused an apparent decrease in the proportion of microglia. Representative data from 1 out of 2 independent experiments are shown.

Based on histological findings, we employed flow cytometry to quantify the immune cells infiltrating the infected nervous tissue to see the dynamics of the cellular immune response. Apart from the spinal cord, we also examined the brain stem, the cerebellum, and the hemispheres, which can also be invaded by schistosomula, however, to a much lesser extent (Fig 2E). The spinal cord and hemispheres were infiltrated mainly by eosinophils, lymphoid cells, and macrophages/monocytes with a peak 14 dpi (Fig 5A and 5D). A similar situation was observed in the brain stem (Fig 5B), where a significant increase was also noticed in microglia and neutrophil counts 7 dpi. In the cerebellum, eosinophil counts spiked 14 dpi (Fig 5C), but neutrophil, macrophage/monocyte, and lymphoid cell counts reached climax already 7 dpi. The microglia counts remained unchanged in all segments, except for the brain stem 7 dpi (see above). However, their relative representation stooped in favor of infiltrating leukocytes, especially eosinophils (Fig 5E–5H). Taken together, *T*. *regenti* neuroinvasion led to striking infiltration of the CNS with peripheral leukocytes. Eosinophils undoubtedly prevailed in all examined segments having a peak 14 dpi.

### Infected spinal cords displayed transcriptional upregulation of immune system pathways and disruption of neurophysiological functions

**Fig 6:**
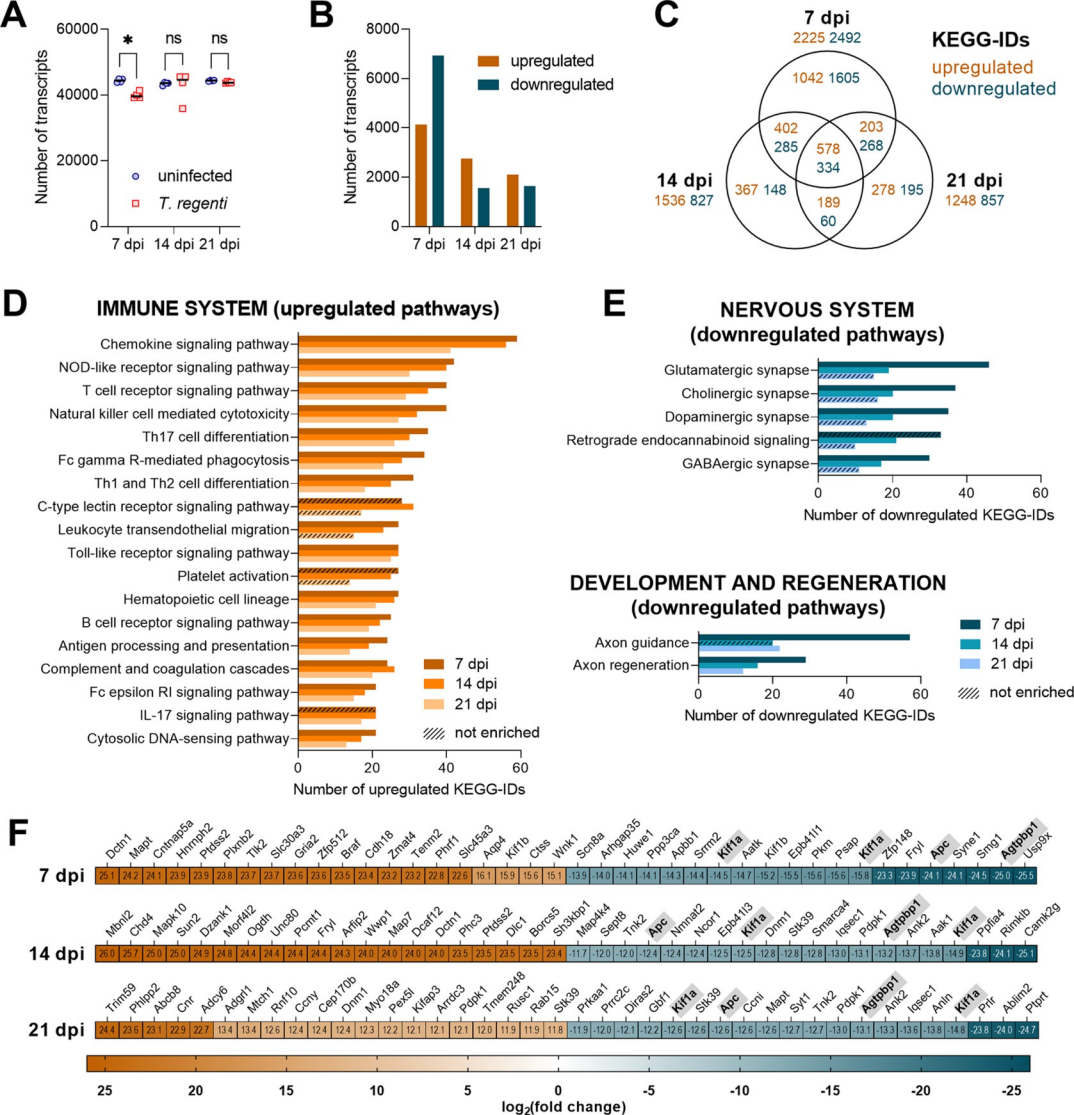
Transcriptomic analysis of the spinal cord of *T*. *regenti*-infected mice. **(A)** The number of transcripts identified in the spinal cords of infected or age-matched uninfected (control) mice 7, 14, and 21 dpi. The transcriptomic analysis was performed with a group of mice (n = 4 per each time point) infected independently from other experiments presented herein. Data were evaluated by ordinary 2-way ANOVA and Šidák’s test; *p<0.05. **(B)** The number of differentially expressed transcripts 7, 14, and 21 dpi based on log_2_fold change (FC; upregulated >2, downregulated <-2). **(C)** Differentially expressed transcripts with Kyoto Encyclopedia of Genes and Genomes (KEGG) annotation and their exclusive/shared occurrence in the infected spinal cord 7, 14, and 21 dpi. **(D)** Enriched immune system pathways of upregulated transcripts according to KEGG annotation. **(E)** Enriched pathways of downregulated transcripts linked with the nervous system and development and regeneration according to KEGG annotation. **(F)** Top 20 up-/downregulated transcripts (based on log_2_FC) 7, 14, and 21 dpi. Abbreviated gene names are shown, gray background marks genes found among the “top 20” at all time points.

To get a complex insight into the processes ongoing in the infected spinal cords, transcriptomic analysis of the whole tissue was performed 7, 14, and 21 dpi using respective age-matched uninfected controls. In total, 24 transcriptomes were sequenced with an average number of 43,115 ± 2,276 transcripts (Fig 6A). RNA-seq read data are available for download *via* the NCBI sequence read archive (BioProject ID: PRJNA716607). Differential expression analysis showed the highest number of differentially expressed transcripts (DETs) 7 dpi when more transcripts were downregulated (6,943) than upregulated (4,128). The number of DETs decreased 14 and 21 dpi, and upregulated transcripts prevailed over downregulated ones (Fig 6B and S1 Table).

**Fig 7:**
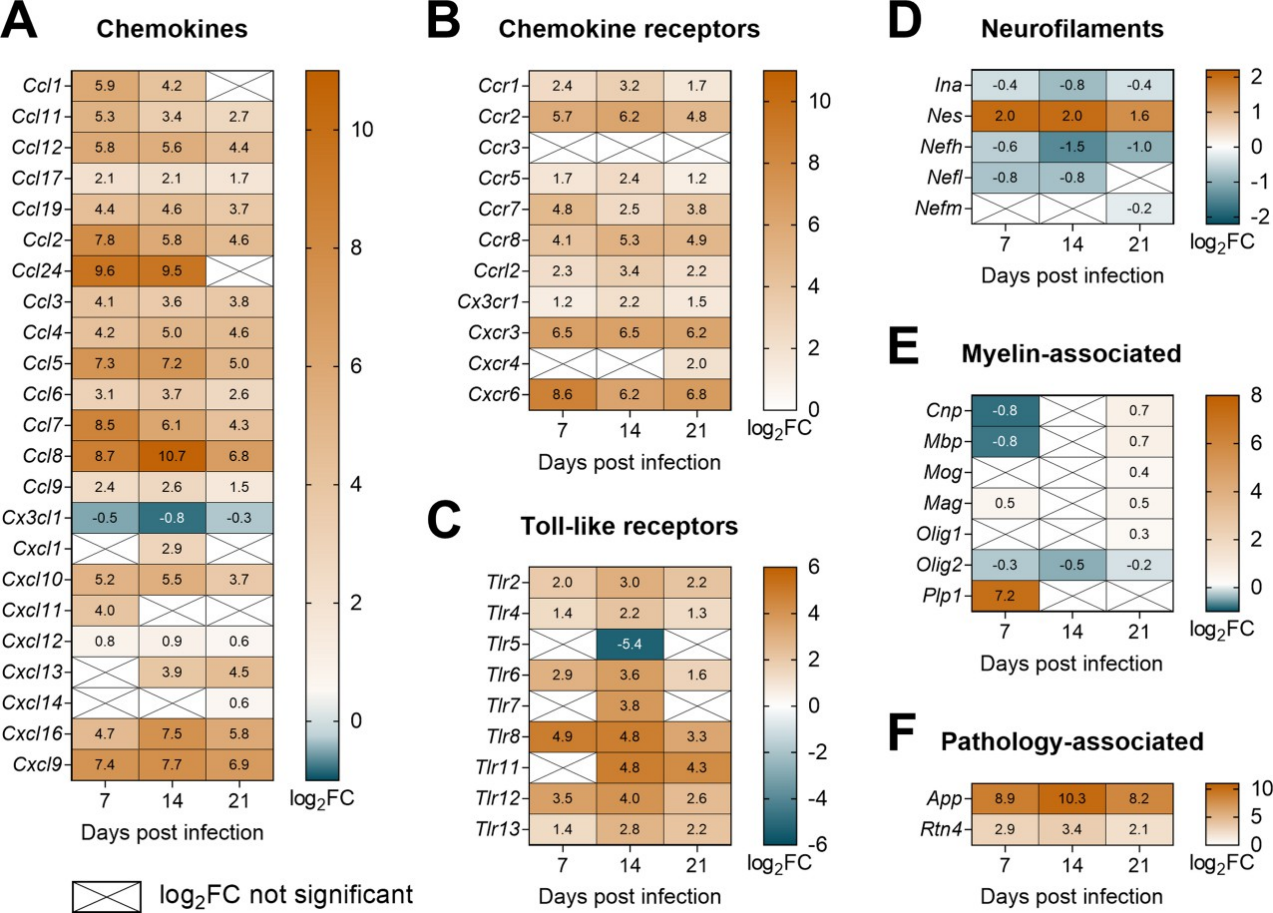
Expression of immune and neural markers in the spinal cord of *T*. *regenti*-infected mice. **(A–F)** The heatmaps show log_2_fold change (FC) in the expression of chemokines (A), chemokine receptors (B), Toll-like receptors (C), neurofilaments (D), myelin-associated markers (E), and pathology-associated markers (F). Genes/transcripts with log_2_FC >2 or <–2 were considered as upregulated or downregulated, respectively. Only genes with a significant log_2_FC are shown unless indicated otherwise (crossed cells). Spinal cords of 4 infected and 4 uninfected age-matched mice were analyzed at each time point.

Kyoto Encyclopedia of Genes and Genomes (KEGG) database was used to annotate DETs to get a general overview of the up-/downregulated processes. In total 2,225, 1,536, and 1,248 upregulated KEGG-IDs were identified 7, 14, and 21 dpi, respectively (Fig 6C), and represented 27 (7 dpi), 47 (14 dpi), and 27 (21 dpi) significantly enriched biological pathways (S1 Table). The most enriched pathways were linked to the immune system at all time points (Fig 6D). For example, chemokine signaling and leukocyte transendothelial migration pathways were significantly enriched 7 and 14 dpi when the spinal cord infiltration by peripheral leukocytes was on the rise (Fig 5A). A detailed analysis revealed the upregulation of several chemokines (*Ccl2*, *Ccl5*, *Ccl7*, *Ccl8*, *Ccl24*, *Cxcl9*, *Cxcl16*) and their receptors (Fig 7A and 7B) which facilitate the recruitment of leukocytes into the CNS. Throughout the infection, remarkable enrichment was also noticed in the Toll-like receptor signaling pathway, which participates in recognizing pathogen-associated molecular patterns. Namely, *Tlr8*, *Tlr11*, and *Tlr12* exhibited the highest upregulation (Fig 7C). Pathways associated with antigen processing were also enriched, especially 7 dpi. Indeed, cathepsin S (*Ctss*), a lysosomal enzyme critical for antigen presentation, was found among the top 20 upregulated DETs 7 dpi (Fig 6F) and retained the markedly elevated expression also 14 and 21 dpi (log_2_fold change = 15 and 11, respectively). Accordingly, Th1/Th2/Th17 cell differentiation and B cell receptor signaling pathways were upregulated as soon as 7 dpi, suggesting early adaptive response activation. These transcriptomic data correspond to our experimental results demonstrating the production of parasite-specific antibodies (Fig 2B) or mixed Th1/Th2/Th17 splenic phenotype [29] 7 dpi. Interestingly, complement and coagulation cascades and the C-type lectin receptor pathway were mostly enriched 14 dpi when the highest eosinophil infiltration and first injured schistosomula were found in the spinal cord (Fig 4C and 4D).

The infection of the mouse spinal cord was also linked with significant downregulation of transcription. Overall, 2,492, 827, and 857 downregulated KEGG-IDs were identified 7, 14, and 21 dpi, respectively (Fig 6C), and represented 33 (7 dpi), 18 (14 dpi), and 9 (21 dpi) enriched pathways (S1 Table). They were mostly associated with signal transduction, synaptic transmission, and axonal development (Fig 6E). Accordingly, we observed enormous downregulation of kinesin family members *Kif1a* and *Kif1b*, essential players in the axonal transport of synaptic vesicles and mitochondria [30,31], and cytosolic carboxypeptidase 1 (*Agtpbp1*, also known as *Ccp*) deficiency of which causes coordination deficits [32]. They all were even among the top 20 downregulated DETs at all time points (Fig 6F). On the contrary, the expression of structural neurofilaments remained almost unchanged except for nestin (*Nes*, Fig 7D) required for glial scar formation [33,34]. Of myelin-associated transcripts, only proteolipid protein 1 (*Plp1*) was strikingly upregulated 7 dpi (Fig 7E), which might boost the activation of microglia [35]. Importantly, no transcriptional evidence was found for demyelinating processes (Fig 7E), which accords with previous histopathological studies [22,26]. However, massive upregulation was observed in the case of the amyloid precursor protein (*App*, Fig 7F) at all time points, which is likely linked to axonal injury caused by migrating schistosomula [22].

Altogether, the transcriptional analysis of *T*. *regenti*-infected spinal cords substantially elucidated general features of the host-parasite relationship. Most importantly, we demonstrated upregulation of immune processes and downregulation of neurophysiological pathways. Based on the transcriptomic data, we further examined several aspects of *T*. *regenti* neuroinvasion.

### Arginase-1 dominated among the proteins found exclusively around the migrating schistosomula, which unselectively fed on the nervous tissue

**Fig 8:**
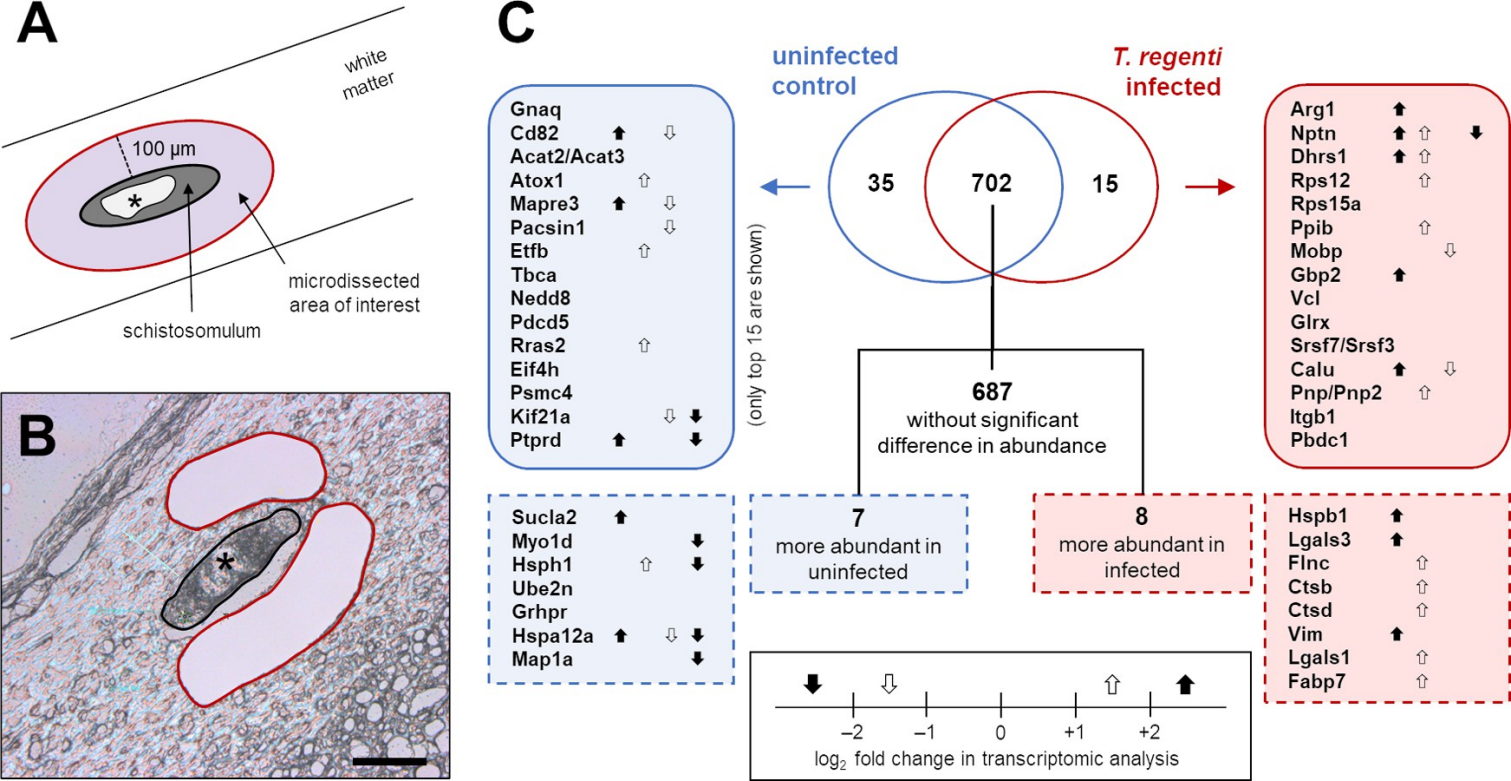
Proteomic analysis of the spinal cord tissue around *T*. *regenti* schistosomula. **(A)** A schematic representation of the region of interest. The area within 100 μm from the schistosomulum was microdissected as well as the schistosomulum digestive tract (marked by an asterisk; for intestinal results see the main text). **(B)** A real image from the microscope, labeling is consistent with (A)–i.e., the already microdissected area is outlined in red. Scale bar = 100 μm. **(C)** A summary of the proteomic analysis of the microdissected nervous tissue. Proteins exclusively found (thick line) or more abundant (dashed line) in uninfected (blue) or *T*. *regenti*-infected (red) spinal cords are shown. The up/down arrows indicate a log_2_fold change (FC) in the expression of the protein mRNA as revealed by the transcriptomic analysis (see Fig 6). More arrows per one protein mean that more isoforms were detected in the transcriptome. If no arrow is shown, the log_2_FC was negligible (–1; +1) or not significant. The proteomic analysis was performed with samples obtained from a group of mice (n = 3–4, see Materials and Methods for details) infected independently from other experiments presented herein.

To better understand the biological processes in the close vicinity of the migrating schistosomula, we microdissected the surrounding tissue within 100 μm from the schistosomula (Fig 8A) and subjected it to the mass spectrometry (MS) analysis. Infected spinal cords were analyzed only 7 dpi as the highest number of schistosomula can be found at this time point and it is possible to properly distinguish the schistosomula-host tissue interface (Figs 6B and 8B). Also, most DETs were found at this time point. Initially, microdissects from three spinal cord segments (sacral, lumbar, thoracic) were analyzed separately. As no significant differences were found in the protein composition of microdissects from different segments (S2 Table, sheets F–H), the subsequent data analysis was performed on merged datasets from all three segments.

Altogether, 1,690 proteins were identified in infected and uninfected (control) spinal cords. In total, 752 proteins were recorded in at least 3 out of 4 replicates and therefore were considered reliably identified. The vast majority (705) of proteins were common to microdissects from infected and control spinal cords, but 15 of them were differentially abundant in infected (8) or control (7) ones (Fig 8C, dashed line outline and S2 Table, sheet C). Fifteen proteins were exclusively found in the microdissects from infected spinal cords (Fig 8C, thick red outline and S2 Table, sheet A), including those presumably involved in the host immune response: arginase (a marker of alternatively activated M2 microglia/macrophages), integrin beta 1 (leukocyte adhesion molecule), or interferon-induced guanylate-binding protein 2 (enigmatic protein with antiparasitic activity; [36]). On the contrary, 35 proteins were exclusively found in uninfected mice (Fig 8C, thick blue lines and S2 Table, sheet B). They were linked mainly with the cytoskeleton and cell signaling, but no specific pathway/pattern was recognized. Corroborating with the proteomic data, most exclusive and differentially abundant proteins were identified as differentially expressed (either up- or downregulated) in the “7 dpi” transcriptome. Some of them were represented by multiple transcription variants differing in the level of expression (Fig 8C, up/down arrows and S2 Table, sheet E).

Additionally, the protein composition of the schistosomula digestive tract was analyzed by MS to reveal the parasite diet. A better understanding of *T*. *regenti* eating habits might help to unveil the host-parasite interactions. Out of 237 reliably identified proteins, 179 were annotated as mouse proteins, 39 as *T*. *regenti*, and 19 shared homology with both organisms (S2 Table, sheet I). The most abundant among mouse proteins were those associated with myelin, such as myelin basic protein, myelin proteolipid protein, 2,3-cyclic-nucleotide 3-phosphodiesterase, and myelin-oligodendrocyte glycoprotein. Furthermore, neuronal and astrocytic intermediate filaments were present (neurofilament light and medium polypeptides, glial fibrillary acidic protein, vimentin) as well as the CNS extracellular matrix glycoprotein periostin. Interestingly, the schistosomula digestive tract also contained proteins related to the host immune system like lysozyme C-1, arginase, or eosinophil cationic protein 1. Endogenous *T*. *regenti* proteins were represented mainly by digestive peptidases (cathepsins B1.6, B1.3, B1.4, C, and leucine aminopeptidase), detoxification enzymes (thioredoxin, peroxiredoxin, and glutathione S-transferase), and metabolic enzymes (lactate dehydrogenase A and glyceraldehyde 3-phosphate dehydrogenase). Proteins homologous to both mouse and *T*. *regenti* included highly conserved proteins such as actin, tubulin, calmodulin, and histones.

Collectively, the proteomic analysis of tissue microdissects demonstrated that markers suggesting activation of the host immune response were present around the schistosomula already 7 dpi. Arginase was the most abundant exclusive protein indicating the presence of microglia/macrophages with the M2 phenotype. We also found that migrating schistosomula are not selective in the diet as a mixture of proteins associated with the nervous tissue and the host immune system was detected in their digestive tract.

### *T*. *regenti* infection induced M2 polarization of microglia/macrophages which accumulated around the migrating schistosomula

**Fig 9:**
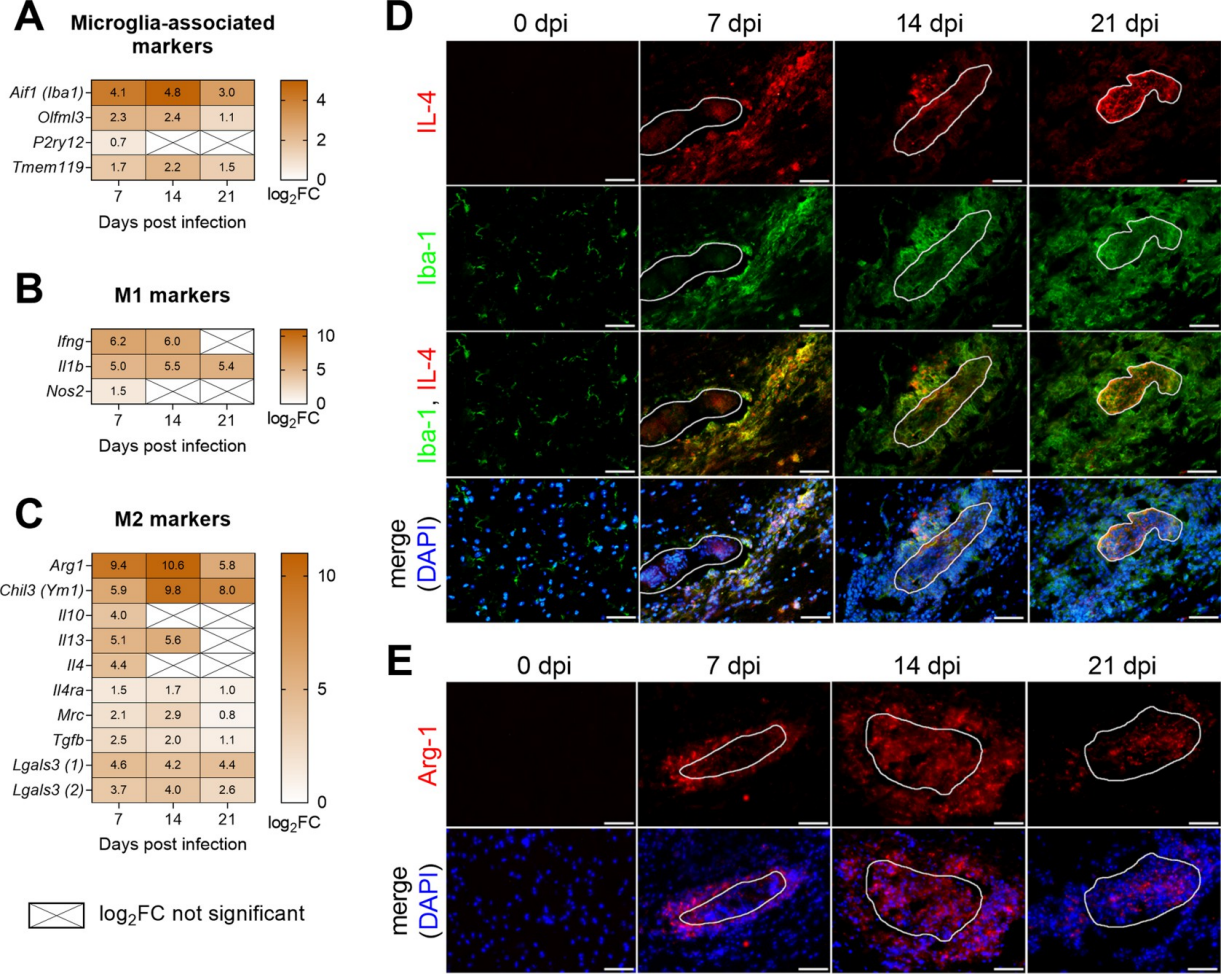
M1/M2 polarization in the spinal cord of *T*. *regenti*-infected mice. **(A–C)** The heatmaps show log_2_fold change (FC) in the expression of markers associated with microglia (A), M1 (B), and M2 (C) polarization. Genes/transcripts with log_2_FC >2 were considered upregulated, and only genes with a significant log_2_FC are shown unless indicated otherwise (crossed cells). Spinal cords of 4 infected and 4 uninfected age-matched mice were analyzed at each time point. **(D–E)** Immunolocalization of IL-4+Iba-1 (D), and Arg-1 (E). Representative images are shown, the white line indicates the space occupied by the schistosomulum. Scale bar = 50 μm.

Polarization of activated microglia/macrophages, either pro-inflammatory M1 or anti-inflammatory M2, largely determines their role in the host immune response. Marked upregulation of *Aif1* (also known as *Iba1*), the marker of microglia/macrophages [37,38], indicated activation of the cells in response to the spinal cord invasion by *T*. *regenti* (Fig 9A). Initially, Iba-1 was mostly detected in the schistosomula migratory tracks (7 dpi), but later, it accumulated around schistosomula (14 dpi) or even within their damaged bodies (Fig 9D). As this observation suggested the active participation of microglia/macrophages in the schistosomula elimination, we further examined the expression of markers related to M1/M2 polarization. Despite upregulation of M1-promoting cytokines (*Ifng*, *Il1b*) throughout the infection, inducible NO synthase (*Nos2*), the effector molecule of M1 polarization, exhibited none or negligible changes in expression (Fig 9B). It corroborates our previous immunohistochemical data demonstrating the lack of inducible NO synthase in the infected spinal cord [26].

On the other hand, we recorded striking upregulation of M2-associated markers, such as arginase (*Arg1*) and chitinase-like 3 (*Chil3*, also known as *Ym1*). Accordingly, M2-triggering cytokines (*Il4*, *Il10*, *Il13*, *Tgfb*) were upregulated 7 and/or 14 dpi along with M2-promoting galectin-3 (*Lgals3*) [39] (Fig 9C). To validate the transcriptomic data, we proceeded with the immunohistochemical detection of IL-4, which drives M2 polarization and the production of arginase [40]. IL-4 colocalized with Iba-1 in the schistosomula migratory tracks 7 dpi but diminished 14 dpi (Fig 9D). However, a strong IL-4 signal was detected in the area of the destroyed schistosomula 21 dpi. On the contrary, arginase (Arg-1) closely surrounded the schistosomula 7 dpi (Fig 9E), which agreed with the previously shown microdissection data (Fig 8C). At 14 dpi, the Arg-1 signal spread further into the tissue but accumulated mostly within the damaged schistosomula 21 dpi.

Collectively, transcriptomic and immunohistochemical data confirmed a strong M2 polarization of microglia/macrophages in the infected spinal cord, especially in the close vicinity of the schistosomula.

### 3D-imaging revealed the expansion of MHC II+ cells during *T*. *regenti* neuroinvasion

**Fig 10:**
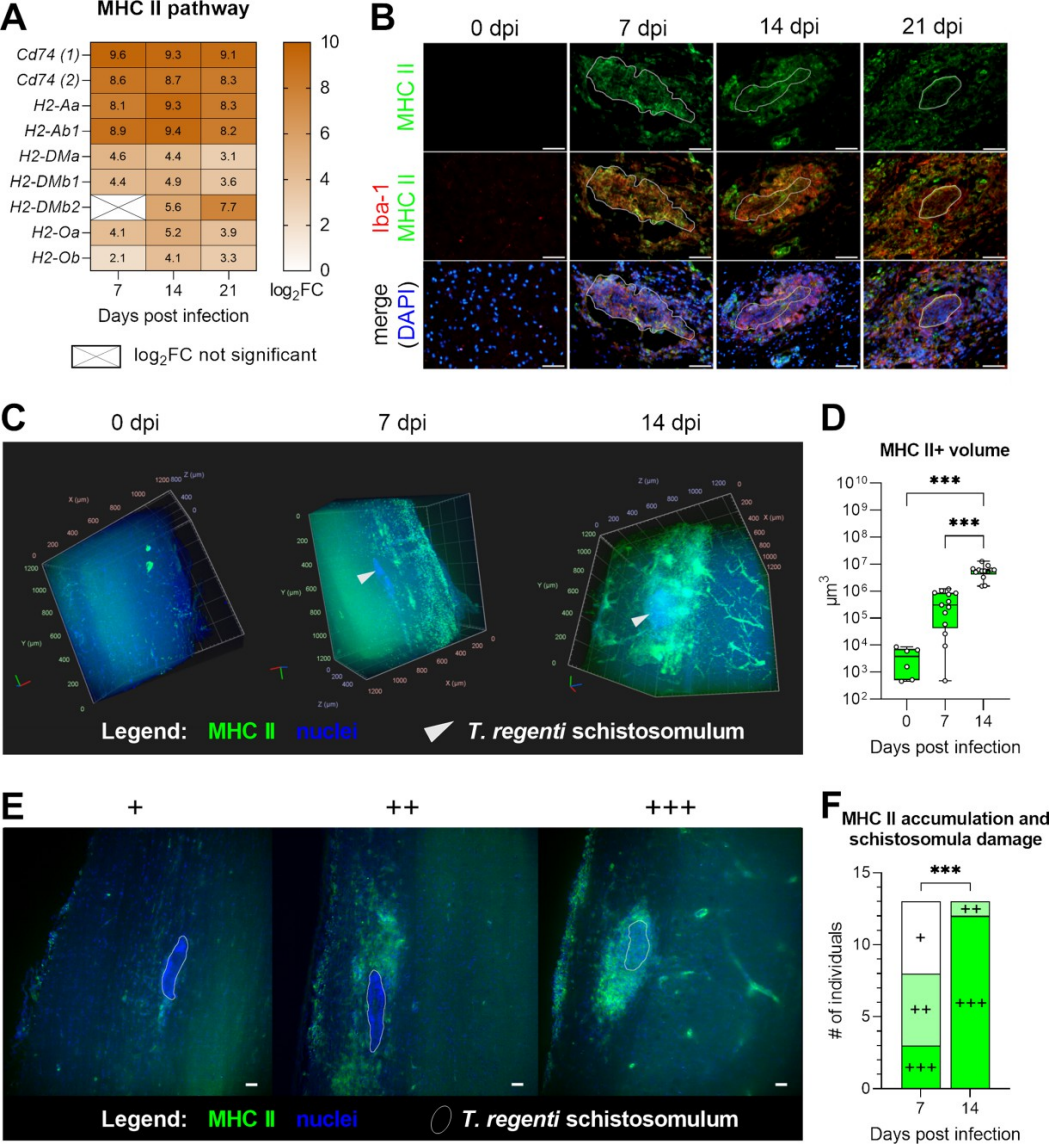
Major histocompatibility complex (MHC) II in the spinal cord of *T*. *regenti*-infected mice. **(A)** The heatmaps show log_2_fold change (FC) in the expression of markers associated with the MHC II pathway. Genes/transcripts with log_2_FC >2 were considered upregulated, and only genes with a significant log_2_FC are shown unless indicated otherwise (crossed cells). Spinal cords of 4 infected and 4 uninfected age-matched mice were analyzed at each time point. **(B)** Immunolocalization of MHC II and Iba-1. Representative images are shown, the white line indicates the space occupied by the schistosomulum. Scale bar = 50 μm. **(C)** 3D images obtained by light-sheet fluorescence microscopy (LSFM) showing MHC II+ cells in the spinal cord. Schistosomula are marked with grey arrowheads. **(D)** Quantification of MHC II+ volume around schistosomula. It was computed in the virtual box around the schistosomula (400×800×110 μm, S5 Video) or six randomly chosen areas in healthy mice. **(E)** Patterns of MHC II accumulation and its relation to schistosomula damage. Three categories were recognized: (+) very few MHC II+ cells around/behind intact schistosomula, (++) noticeable infiltration of MHC II+ cells enclosing intact schistosomula (but no MHC II signal within the schistosomula), (+++) massive clusters enclosing damaged schistosomula (MHC II signal present also inside the schistosomula). 2D images were obtained by LSFM and created from five stitched planes in the z-axis (1.4 μm each). Scale bar = 50 μm. **(F)** Quantification of MHC II accumulation and schistosomula damage using the categories from (E). Thirteen schistosomula out of two mice were examined at each time point. Data were evaluated by Kruskal-Wallis and Dunn’s test (D) and Fisher’s exact test (F); ***p<0.001.

Expression of major histocompatibility complex (MHC) II, either by professional antigen presenting or other cells, mirrors the activation status of the host immune response. In *T*. *regenti*-infected spinal cords, we recorded striking upregulation of *Cd74*, *H2-Aa*, *H2-Ab1*, and other members of the MHC II pathway throughout the infection (Fig 10A). Immunohistochemistry revealed accumulation of MHC II+ cells around the schistosomula and partial colocalization of MHC II with Iba-1, the marker of microglia/macrophages (Fig 10B). Thanks to the availability of a suitable model (MHC II-EGFP mice), we employed light-sheet fluorescence microscopy (LSFM), which allowed us to display a 3D tissue context and visualize MHC II distribution *in toto* 7 and 14 dpi. High levels of background autofluorescence impeded analysis of the gray matter, so we examined white matter regions of the thoracic spinal cord, which is preferred by the schistosomula anyway [41].

Seven dpi, the MHC II+ cells were scattered in the parenchyma, but some of them already contacted the schistosomula (Fig 10C and S2 and S3 Videos). Massive MHC II+ clusters formed around the schistosomula 14 dpi when the MHC II signal often copied the shape of parenchymal blood vessels and anterior or posterior median sulci (Fig 10C and S4 Video). These observations correlated with data from the volumetric analysis of the MHC II signal, which rocketed 14 dpi (p < 0.0001). Indeed, a 1000-fold increase was recorded in comparison to uninfected mice (Fig 10D). We also attempted to count the exact amount of MHC II+ cells, but the clusters were so dense that single nuclei were hardly recognizable, which hampered the analysis. Nevertheless, the large-scale optical sectioning provided us with a robust insight into the intensity of the immune reaction (seen as MHC II accumulation) around individual schistosomula. We established three categories of the immune reaction (Fig 10E): “mild” (+) with very few MHC II+ cells around/behind (“rocket tail”) the intact schistosomula; “moderate” (++) with noticeable infiltration of MHC II+ cells enclosing the intact schistosomula (but no MHC II signal within the schistosomula); “severe” (+++) with massive clusters enclosing the damaged schistosomula (MHC II signal present also inside the schistosomula). While mild and moderate reactions prevailed 7 dpi, severe reactions dominated 14 dpi (Fig 10F; p = 0.0009).

Altogether, transcriptomic analysis and 2D/3D imaging demonstrated a strong upregulation of the MHC II pathway in *T*. *regenti*-infected spinal cords. Interestingly, the MHC II occurred not only around the migrating schistosomula but also along the blood vessels and within the meningeal areas indicating extensive neuroinflammation.

### *T*. *regenti* infection affected vascular permeability, blood-brain barrier integrity and triggered reactive astrogliosis

**Fig 11:**
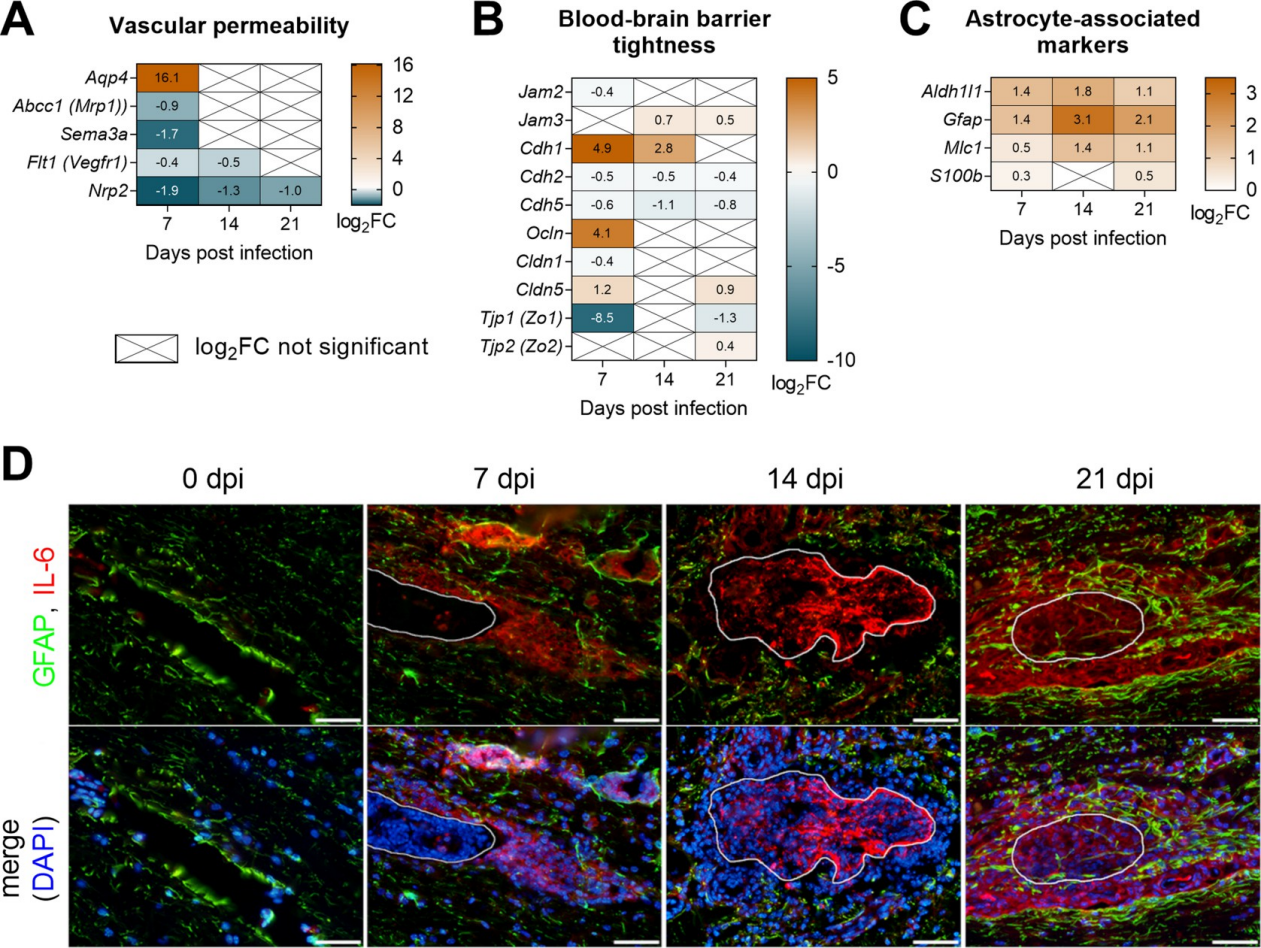
Pathology associated with *T*. *regenti* migration within the mouse spinal cord. **(A–C)** The heatmaps show log_2_fold change (FC) in the expression of markers associated with vascular permeability (A), blood-brain barrier tightness (B), and astrocytes (C). Genes/transcripts with log_2_FC >2 or <-2 were considered upregulated or downregulated, respectively. Only genes with a significant log_2_FC are shown unless indicated otherwise (crossed cells). Spinal cords of 4 infected and 4 uninfected age-matched mice were analyzed at each time point. **(D)** Immunolocalization of astrocytic glial fibrillary acidic protein (GFAP) and astrogliosis-promoting IL-6 demonstrated the most prominent astrocyte hypertrophy 21 dpi. Representative images are shown, the white line indicates the space occupied by the schistosomulum. Scale bar = 50 μm.

Schistosomula migration within the CNS might cause severe pathology. First, we focused on changes in vascular permeability and integrity of the blood-brain barrier, which are critical for maintaining CNS homeostasis. Among markers of vascular permeability, aquaporin-4 (*Aqp4*), the water channel localized to astrocyte endfeet, was strikingly upregulated 7 dpi (Fig 11A). Expression of E-cadherin (*Cdh1*) and occludin (*Ocln*), proteins responsible for tightness of the blood-brain barrier [42], was also increased, especially 7 dpi (Fig 11B). On the contrary, remarkable downregulation was noticed in tight junction protein 1 (*Tjp1*, also known as *Zo1*), a scaffold protein anchoring tight junctions to the actin fibers [42]. Although our transcriptomic data suggested that vascular permeability and blood-brain barrier integrity were mildly altered 7 dpi, we did not observe any significant increase in extravasation of Evans blue into the nervous tissue (S2 Fig).

Second, we examined the tissue injury associated with schistosomula migration. Astrocytic glial acidic fibrillary protein (*Gfap*) was upregulated 14 and 21 dpi (Fig 11C), which pointed to the initiation of reactive astrogliosis and formation of glial scar [43]. While GFAP showed normal distribution within the nervous tissue 7 dpi, it accumulated around schistosomula lesions and in their migratory tracks at later time points (Fig 11D). Astrocyte hypertrophy was most prominent 21 dpi when astrocyte processes enclosed the schistosomula or even grew through the space occupied by them. To support the observation of astrocyte activation, we also immunolocalized IL-6, which promotes astrogliosis and glial scar formation [44]. IL-6 was detected in the schistosomula migratory tracks 7 dpi and in the area of the damaged schistosomula and their surroundings 14 and 21 dpi (Fig 11D). These data indicated that reactive astrogliosis and glial scar, presumably driven by IL-6, developed to repair the tissue injured by schistosomula migration.

### DNA fragmentation occurred at the host-schistosomulum interface, but schistosomula antigens did not trigger apoptosis in neurons or glial cells

**Fig 12:**
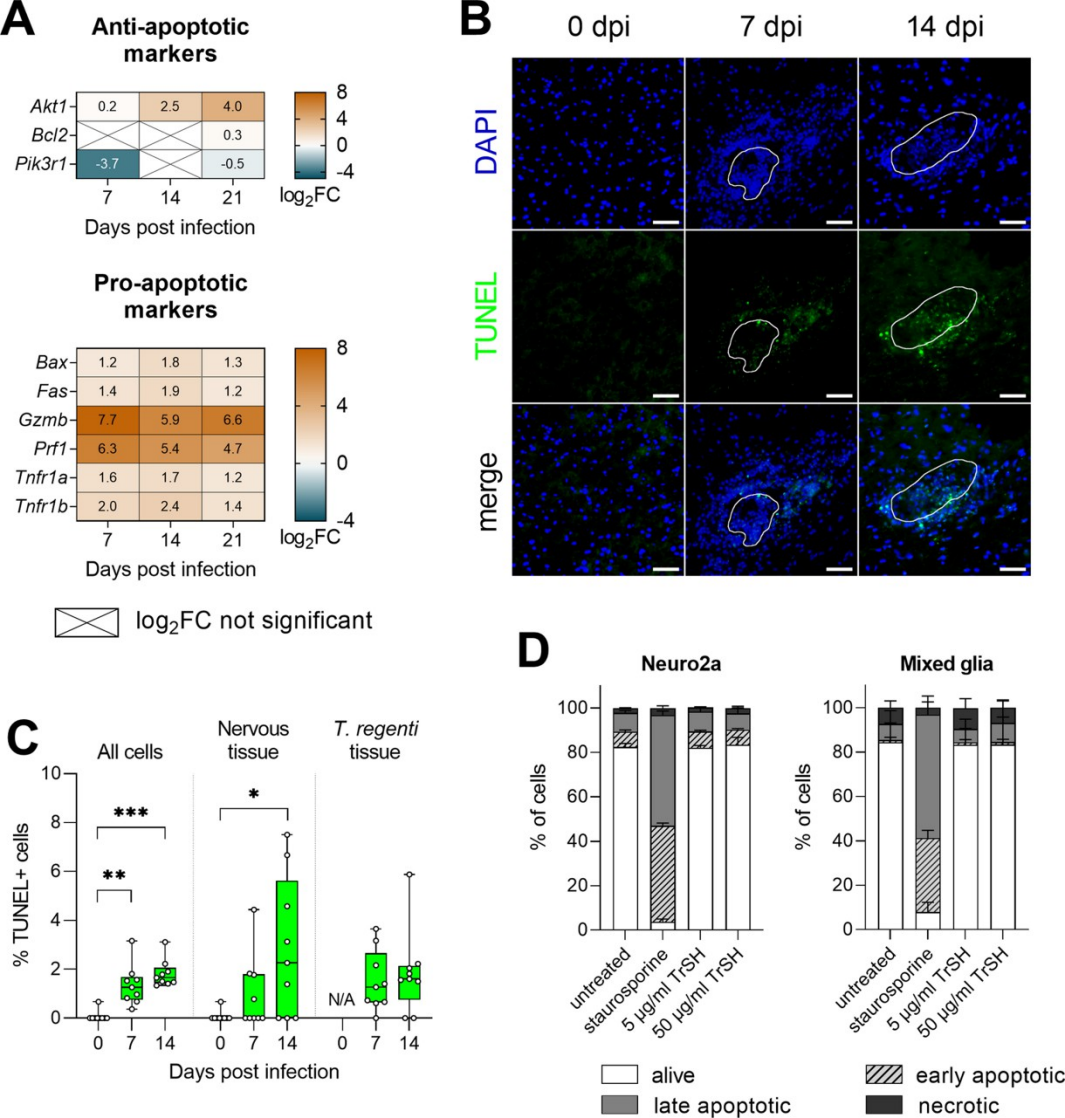
Apoptosis in the spinal cord of *T*. *regenti*-infected mice. **(A)** The heatmaps show log_2_fold change (FC) in the expression of pro- and anti-apoptotic markers. Genes/transcripts with log_2_FC >2 or <–2 were considered as upregulated or downregulated, respectively. Only genes with a significant log_2_FC are shown if not indicated otherwise (crossed cells). Spinal cords of 4 infected and 4 uninfected age-matched mice were analyzed at each time point. **(B)** Detection of DNA fragmentation (TUNEL+ cells) in the spinal cord cryosections. Representative images are shown, the white line indicates the space occupied by the schistosomulum. Scale bar = 50 μm. **(C)** Quantification of TUNEL+ cells in the spinal cord cryosections shown in (B). The frequency of TUNEL+ cells is shown among all cells or separately for the host nervous tissue and *T*. *regenti* tissue. **(D)** Detection of apoptotic populations in Neuro2a and mixed glial cultures treated by *T*. *regenti* schistosomula homogenate (TrSH) for 48 hours; staurosporine was used as a positive control. Pooled data from 4 experiments are shown. Data (C, D) were evaluated by Kruskal-Wallis and Dunn’s test; *p<0.05, **p<0.01, ***p<0.001.

Apoptosis plays an important role in host-parasite interactions as it can be responsible for tissue pathology or help parasites evade the host immune system. In *T*. *regenti*-infected spinal cords, we observed upregulation of pro-apoptotic genes *Gzmb* and *Prf* coding for granzyme B and perforin-1, respectively (Fig 12A). They are expressed by cytotoxic T lymphocytes and natural killer cells which employ them to induce apoptosis in target cells [45]. Therefore, we stained fragmented DNA, a hallmark of apoptosis, by the TUNEL assay to localize the apoptotic cells within the infected spinal cord. While no TUNEL+ cells were found in the uninfected spinal cord, their frequency increased 7 and 14 dpi, both in host and schistosomula tissues (Fig 12B and 12C). The TUNEL+ cells were localized especially at the host-parasite interface, but some were also recorded in the schistosomula migratory tracks. To assess the possibility that schistosomula-derived molecules directly induce apoptosis of the host neural cells, we stimulated mouse Neuro2a and primary mixed glial cultures with a soluble fraction of schistosomula homogenate. However, the treatment did not alter the frequency of apoptotic populations (Fig 12D). These data suggested that apoptosis was not a major feature of *T*. *regenti* neuroinvasion and was not responsible for tissue pathogenesis.

## Discussion

Apart from being a causative agent of human cercarial dermatitis [46,47], *T*. *regenti* invades the CNS of experimentally infected mice [19,20]. However, neuroinvasion by this species has not been reported from humans. Here we thoroughly investigated the invasion of the mouse CNS by *T*. *regenti* to better understand the diversity of host-parasite interactions during neuroinfections. Although *T*. *regenti* does not fully recapitulate any human neuroinfection, it represents a well-characterized and easily trackable system, providing novel insights into the diversity of pathogen-related neuroinflammation.

Of the mouse CNS, *T*. *regenti* predominantly affects the spinal cord to where it migrates from the skin *via* peripheral nerves [19–22,41]. Such a route is extraordinary among neurotropic helminths, which mostly rely on hematogenous spread into the CNS [48]. Indeed, *T*. *regenti* intraneural pathway rather resembles that of the neuropathogenic amoeba *Naegleria fowleri* [49]. Corroborating with the previous studies [19–22], we observed the highest schistosomula burden in the spinal cord 7 dpi. In addition, we detected *T*. *regenti* DNA in some of the hemisphere samples 14–21 dpi, but we assume that it originated from a few and most likely already damaged schistosomula since viable parasites were only rarely isolated even 7 dpi. Largely restricted access of schistosomula to the brain could explain the lack of working memory deficit, anxiety, or depression-like behavior, which stem from dysfunction of hemisphere centers [50–52].

By affecting mostly the spinal cord, *T*. *regenti* differs from other neurotropic helminths, such as *Angiostrongylus cantonensis*, *Toxocara* spp., or *Taenia solium*, which primarily invade the brain and impair cognitive functions or cause seizures [9,53–56]. Nevertheless, *T*. *regenti* resembles the neurotropic larvae of *A*. *cantonensis* and *Toxocara* spp. regarding size and active migration within the nervous tissue [9,54]. On the contrary, passive dissemination of schistosome eggs into the spinal cord induces human neuroschistosomosis, which often manifests as myelitis [57]. Despite sharing some histopathological or clinical features, such as myelitis and motor dysfunction of lower/hind limbs [58], *T*. *regenti* neuroinvasion cannot be directly compared to neuroschistosomosis, mainly due to the different nature of the causative agents (migrating schistosomula vs. disseminated eggs). As a whole, *T*. *regenti* neuroinvasion represents a unique biological phenomenon, but in particular regards, it can be compared to other neurotropic helminths. Regarding the general features, such as size and behavior, *T*. *regenti* represents a relevant comparative model especially for studying the pathology related to the migration of neurotropic helminths such as *A*. *cantonensis* or *Toxocara* spp.

Our complex approach revealed a serious impact of *T*. *regenti* neuroinvasion on the well-being of mice. They manifested with the reduced gain in body weight despite unchanged feed consumption or locomotion. We speculate that this could be explained by increased energy demands related to fighting the infection [59–61]. Of note, a decreased body weight was observed at all time points. It indicates that the peripheral immune response, with a peak 7 dpi [29], and the later neuroinflammation could have affected the weight gain. Reduction of body weight accompanies mouse infections with *A*. *cantonensis* and *Toxocara* spp. as well and often correlates with impaired neurological scores [55,62,63]. These observations highlight the systemic impacts of helminth neuroinfections, however, direct evidence linking the weight loss with presumably increased energy consumption related to the host immune reaction is missing.

Extensive behavioral testing revealed deficits in lower body motor functions in *T*. *regenti*-infected mice. Up to now, mainly ducks (definitive hosts) and immunocompromised mice were reported to suffer from neuromotor disorders related to *T*. *regenti* neuroinvasion [19]. This fact was explained by the higher schistosomula burden in such hosts and hence more frequent axonal injury [21,22]. Similar neuromotor abnormalities are also often present in mice infected with *A*. *cantonensis* and *Toxocara* spp. [53,62,64,65]. Dysregulated expression of myelin-associated genes, extensive tissue demyelination, and neuronal apoptosis are believed to be the pathological basis of such behavioral changes [63,65–68]. However, we detected no remarkable apoptosis or downregulation of myelin-associated genes in *T*. *regenti*-infected spinal cords. Also, no loss of myelin was seen in previous histological studies [22,26]. On the contrary, our transcriptomic data unveiled a massive downregulation of neuronal signal transduction, synaptic transmission, and axonal development. For example, we noticed striking downregulation of kinesin superfamily motor proteins *Kif1a* and *Kif1b*, which are essential for anterograde axonal transport [30,31]. Their effect is evident in *Kif1a*- and *Kif1b*-knockout mice, which exhibit reduced motor activity, abnormal limb movements, and impaired balance [69,70]. Therefore, we suggest that the disruption of neurophysiological functions in the spinal cord was responsible for the observed motor deficits. Such wide-range downregulation of neuronal functions has so far not been noticed in other transcriptomic studies focusing on helminth neuroinfection [65,66] but was recently reported from mouse brains infected with *Toxoplasma gondii* [7]. Specifically, Boillat *et al*. propose that behavioral alterations caused by *T*. *gondii* reflect side effects of neuroinflammation, i.e., the neuro-immune response plays a major role in modulating host behavior [7]. Therefore, we suggest that impairment of neurophysiological functions, potentially triggered by neuroinflammation, might be a novel pathological mechanism also in helminth neuroinfections.

Contrary to ducks, mice as accidental (dead-end) hosts efficiently control *T*. *regenti* neuroinvasion. This makes them apt species for studying the protective immune response. Moreover, the lack of well-defined duck strains and a suitable toolbox (mainly antibodies) markedly limits the immunological studies on *T*. *regenti* in ducks anyway. Our complex data strongly support the view that rapid activation of the mouse immune response leads to the elimination of schistosomula within 2–3 weeks post infection. It agrees with historical records of schistosomula suspended growth and reduced survival in immunocompetent mice [21,22,71]. Such immune-mediated host incompatibility is also observed in the case of *A*. *cantonensis*–it flourishes in rats but is retarded in mice. The latter develop much stronger neuroinflammation than rats, which is pronounced after the death of migrating larvae [9,11,72,73]. On the contrary, mice are suitable paratenic hosts for *Toxocara* spp., which are well adapted to survival in their tissues, including the CNS [74]. Similarly, viable cysticerci of *T*. *solium* actively evade the human immune response and cause no or mild inflammation [56]. Hence, *T*. *regenti* in mice is a perfect model enabling us to study natural mechanisms leading to helminth elimination within the CNS.

A strong eosinophilic meningomyelitis was the most prominent histopathological hallmark of *T*. *regenti* neuroinvasion. Comparison of peaks in eosinophil counts in the peripheral blood (7 dpi) and the spinal cord (14 dpi) demonstrated spatiotemporal separation of the eosinophil-based immune response in the periphery and within the CNS. A similar distinction was also observed in mice infected with *A*. *cantonensis* [73]. Recruitment of eosinophils into the *T*. *regenti*-infected spinal cords, found in our study, was presumably mediated mainly by eotaxin-2 coded by *Ccl24* [75], which displayed 180- and 700-fold increased expression 7 and 14 dpi, respectively. However, participation of other eosinophil-attracting chemokines upregulated at these time points (*Ccl5*, *Ccl7*, *Ccl8*) cannot be excluded and is even likely. Moreover, elevated expression of *Chil3* could have boosted maintenance of the tissue eosinophilia [76], especially 14 dpi. It was suggested for *A*. *cantonensis*-infected mice that Chil3 aggravates eosinophilic meningitis *via* a positive feedback loop mediated by IL-13 [73] which was also markedly upregulated in our samples. Our data collectively demonstrate a significant infiltration of eosinophils into the *T*. *regenti*-infected spinal cords, suggesting their prospective role in eliminating schistosomula.

Eosinophils harm helminths by antibody- and/or complement-induced secretion of toxic granule proteins and reactive oxygen species [77]. The role of eosinophils in limiting the parasite burden was demonstrated in murine neurocysticercosis [78], but specific effector mechanisms employed to combat helminths have not been defined. As our dissection and histological data indicated elimination of schistosomula around 14 dpi, we searched the transcriptomic datasets for pathways related to eosinophil effector functions upregulated/enriched mostly at this time point. Interestingly, we found that the complement and coagulation cascades were enriched. Specifically, components of C1 complex and C2, C3, C4, and C5 complement components were significantly upregulated (log_2_fold change >2), suggesting activation of the classical complement pathway initiated by antigen-antibody complexes. This mechanism is known to trigger eosinophil degranulation leading to killing schistosomula of *S*. *mansoni* [79] and we propose that it harms *T*. *regenti* schistosomula within the CNS in the same manner.

Eosinophil extracellular DNA traps (EETs) represent another component of the antimicrobial arsenal. Being released in the C-lectin-dependent manner, the EETs can immobilize helminths and facilitate their further clearance, as recently demonstrated in the case of nematode microfilariae [80]. In *T*. *regenti*-infected spinal cords, the C-lectin receptor signaling pathway was mostly enriched 14 dpi and, e.g., dectin-1 was upregulated (log_2_fold change >5) even at all time points. Of note, dectin-1 is a C-type lectin functioning as a transmembrane pattern-recognition receptor [81] mediating the release of eosinophil extracellular DNA traps [80]. Our observation raises an exciting question to be further tested of whether EETs are released in *T*. *regenti*-infected spinal cords and if they can halt the actively moving helminths in the CNS.

Microglia, the CNS-resident macrophage-like cells, are vital players in the CNS innate immunity as they sense and eliminate invading pathogens [82]. Therefore, they were implicated as the cells initiating the neuroinflammation in *A*. *cantonensis*- or *T*. *regenti*-infected mice [25,83]. Our transcriptomic and 2D/3D imaging data confirmed upregulation of microglia/macrophage markers in the infected spinal cord and continuous accumulation, even very intimate, of Iba-1+ MHC II+ cells around the schistosomula as soon as 7 dpi. At this time point, we noticed upregulation of *Tlr2*, *Tlr6*, *Tlr8*, and *Tlr12*, which can be expressed by microglia [84]. Of them, the heterodimer of TLR2/TLR6 might participate in recognition of the *T*. *regenti* infection similarly to murine neurocysticercosis [85]. However, the role of TLR8 and TLR12 in the initial schistosomula recognition is expected to be relatively minor as they both are intracellular receptors, and mouse TLR8 is even nonfunctional in terms of immune recognition [86]. Moreover, a C-type lectin-dependent pathway, including dectin-1 as a receptor [87,88], might be involved in detecting glycans, which are abundant on the surface and in the excretory-secretory products of the parasites [89,90]. Phagocytosis by mononuclear cells of glycan-rich material was shown in murine neurocysticercosis and promoted the infiltration of immune cells into the CNS [8,91]. Thus, we conclude that microglia might serve as sensors for *T*. *regenti* in the spinal cord using diverse and complementary recognition pathways.

Beyond recognition, Lichtenbergová *et al*. proposed that activated microglia/macrophages play the main role in destroying *T*. *regenti* schistosomula in the nervous tissue [22]. However, our data show that a strong type 2 cytokine milieu, driven by upregulated *Il4* and *Il13*, was established in the infected spinal cords suggesting induction of the alternatively activated M2 microglia/macrophage [40,92–94]. The transcriptomic M2 signature was confirmed by the detection of arginase, a hallmark of alternatively activated microglia/macrophages, on the protein level. Induction of such environment is typical also for other helminth neuroinfections [95–97] and can be interpreted as helminth-induced prevention of M1 polarization, which is associated with the production of detrimental nitric oxide. Corroborating with this assumption, *T*. *regenti* schistosomula were shown not to trigger nitric oxide production *in vitro* or the spinal cord [25,26]. The role of microglia/macrophages in killing *T*. *regenti* schistosomula thus needs to be significantly reconsidered. We propose that activated microglia/macrophages phagocytose the tissue debris in the schistosomula migratory tracks–similarly to the gitter cells in *Toxocara*-infected brains [54,68]. Later, they might also clean the opsonized remnants of the already damaged schistosomula. Localization of Iba-1+ and/or MHC II+ cells, revealed by 2D/3D imaging techniques, and enriched FcγR-mediated phagocytosis pathway underpins the novel hypothesis on microglia/macrophage functions in *T*. *regenti*-infected spinal cords.

Finally, we suggest that the M2 polarization promotes repair of the injured nervous tissue. Astrocyte-mediated IL-6-driven formation of the glial scar, which we observed 14–21 dpi, is commonly considered the primary mechanism of the nervous tissue repair, also known from other helminth neuroinfections [22,98,99]. However, M2 microglia/macrophages promote tissue regeneration and remyelination by driving differentiation of oligodendrocytes [100,101]. Chil3, expressed and secreted by M2 cells, promotes oligodendrogenesis [102] and arginase helps axon regeneration by synthesizing polyamines, such as spermidine [103,104]. Both *Chil3* and *Arg1* were massively upregulated in *T*. *regenti*-infected spinal cords, and arginase was even the most abundant protein found in the close proximity to the schistosomula. At the same time, no signs of demyelination or remarkable tissue pathology were noticed. Thus, we conclude that induction of M2 polarization within the infected spinal cord limits extensive pathology potentially associated with *T*. *regenti* neuroinvasion. Identification of the parasite molecules responsible for this effect is warranted as they might represent suitable, tissue-specific immunomodulators applicable against autoimmune or neurodegenerative diseases.

In summary, we present a comprehensive, transcriptome-driven study of the schistosome invasion into the mouse spinal cord. We revealed a remarkable downregulation of neurophysiological functions, which contribute to motor deficits observed in infected mice. The host immune response is dominated by eosinophilic inflammation, which eliminates the infection. A strong M2 polarization evidenced, i.a., by substantial production of arginase, presumably prevents extensive tissue damage. Due to parasitological, immunological, or pathological similarities with human neuropathogens, *T*. *regenti* represents a suitable model for further research of neuroinfections.

## Materials and methods

### Ethics statement

Animal experiments were conducted in compliance with the European and Czech legislation (EU Directive 2010/63/EU, Act No 246/1992). Animal welfare committees of the Faculty of Science, Charles University, and the Ministry of Education, Youth and Sports of the Czech Republic approved all experimental procedures (MSMT-33740/2017-2, MSMT-37946/2020-3). Isoflurane anesthesia followed by transcardial perfusion (exsanguination) were employed to sacrifice the mice as indicated below.

### Animals

C57BL/6JOlaHsd mice (Envigo, Venray, Netherlands) were housed in the Centre for Experimental Biomodels (First Faculty of Medicine, Charles University) or the animal housing facility (Faculty of Science, Charles University). The mice were kept in groups of 4–10 individuals/cage (if not stated otherwise) and provided with food and water *ad libitum*; food consumption was monitored on a weekly basis. Infection with *T*. *regenti* cercariae was performed at the age of 8 weeks. Mixed glial cultures were prepared from newborn pups of both sexes, no older than 48 hours. Light-sheet fluorescence microscopy imaging was performed on organ samples from MHC II-EGFP C57BL/6J knock-in mice provided by Prof. Jan Černý (Department of Cell Biology, Faculty of Science, Charles University).

### Parasites and infection of mice

A complete life cycle of *T*. *regenti* is kept at the Department of Parasitology, Faculty of Science, Charles University. Freshwater snails (*Radix lagotis*) and domestic ducks (*Anas platyrhynchos* f. domestica) are used as intermediate and definitive hosts, respectively [19]. Percutaneous infection of female mice was done according to the well-established “water bath” protocol using the infection dose of 2000 freshly collected cercariae [22,26,29,41].

### Experimental design

A comprehensive investigation was carried out to examine the effects of *T*. *regenti* infection on mice, emphasizing the host immune response and pathology in the CNS (Fig 1). To characterize the general/systemic impact of the infection, body and spleen weights were monitored as well as levels of *T*. *regenti*-specific serum antibodies, blood eosinophil counts, and behavioral alterations of infected mice. Host-parasite interactions in the CNS, especially in the spinal cord, were then broadly explored by several approaches summarized in Fig 1. Uninfected mice were always used as a control group, and specific age-matching is shown in Fig 1. The time points at which the analyses were performed represent distinct phases of the infection [22].

### ELISA

*T*. *regenti*-specific IgG1 and IgG2a were analyzed in the serum samples (diluted 1:80) of mice according to [29]. The cut-off value was calculated according to [105] for a 99.9% confidence level.

### Parasite burden in the CNS

Two complementary methods were used to assess *T*. *regenti* burden in the CNS. First, we directly quantified *T*. *regenti* schistosomula actively released from the spinal cord, brain stem, cerebellum, and hemispheres after the organs were torn into 1–2 mm pieces by sharp forceps in phosphate-buffered saline (pH 7.4; PBS) [26]. The organ suspensions were independently inspected by three people within 6 hours which is enough for viable schistosomula to leave the tissue.

Second, we detected schistosomula DNA by a quantitative real-time polymerase chain reaction (PCR) in the spinal cord and hemisphere samples intended for flow cytometry. Specifically, we used the material remaining in the cell strainers after filtration of the cell suspensions (~10 mg, see “Flow cytometry” for details). Total DNA was isolated by Exgene Tissue SV (plus!) (GeneAll; Lisbon, Portugal) using 100 μl of the elution buffer and stored at –20°C. qPCR was then carried out in iQ5 Multicolor Real-Time PCR Detection System (Bio-Rad) employing iQ SYBR Green Supermix (Bio-Rad; Hercules, California, USA). *Trichobilharzia*-specific *Sau3A* repetitive sequence was selected as the molecular target, 5’-GTGACTTGCTACAGGTTGG-3’ (forward) and 5’-GGCAAGCTCGTATACCATTC-3’ (reverse) primers were used [106] to amplify the PCR product of the expected size of 200 bp. Five μl of eluted DNA were added into an individual reaction, and each sample was run in a triplicate. UltraPure DNase/RNase-free dH_2_O (Invitrogen; Waltham, Massachusetts, USA) was included as a negative control, while 10 ng of DNA isolated from *T*. *regenti* cercariae (corresponding to the highest standard, see below) served as a positive control in each run. qPCR amplification was performed as follows: 3 min at 95°C, 40 cycles of 15 s at 95°C, 15 s at 57°C, 15 s at 72°C, and 1 min at 72°C. Quantitative cycle values were obtained, and the final parasite DNA calculation was done according to the standard curve ranging from 10 ng to 1 fg (a series of 10-fold dilution). Melting curves were always checked to ensure the specificity of the reaction. The cut-off value was calculated as the mean plus three standard deviations of samples from uninfected mice.

### Behavioral tests

Several tests were performed and evaluated by blinded researchers to explore various aspects of mice behavior (see Table 1 for details). A detailed description of task procedures can be found in S1 Text. Mice intended for behavioral tests were housed individually. In all the tests, the apparatuses were cleaned with 70% ethanol and dried after each session.

**Table 1:** Behavioral testing battery. The tests are listed by their temporal order.

Test	Behavior assessed	Parameters analyzed	Ref.
Elevated plus maze	anxiety; activity	time spent in the open arms; risk assessment behavior; total number of arm visits; total distance walked; looking down behavior	[107,108]
Open field	locomotor activity; anxiety	total distance walked; time spent in the center of the arena	[108–112]
Beam walking	motor coordination	time to traverse the beam; error score	[109,110,112]
Bar holding	endurance and strength of the forelimbs	tightrope hang time	[111]
Marble burying	anxiety	proportion of marbles buried to 2/3	[113]
Novelty-induced hypophagia	anxiety	amount of eaten oat flakes; number of feces; latency to first tasting	[108]
Y-maze	spatial working memory; activity	proportion of correct triads to all triads (alternation); total number of arm entrances; total distance walked	[114]
Grid test	endurance and strength of the forelimbs and hindlimbs	time to jump/fall	[112,115,116]
Tail suspension	depressive-like behavior	immobility duration	[112,117]
Forced swimming	depressive-like behavior	immobility duration	[117]
Footprint analysis	posture and gait characteristics	forelimb and hindlimb step length and step width	[116]

### Histology

Spinal cords were carefully extracted from isoflurane-anesthetized mice perfused by heparinized (10 IU/ml) PBS and 4% paraformaldehyde (PFA). The tissue was post-fixed in 4% PFA overnight at 4°C, dehydrated, and embedded in paraffin blocks. Five μm thick sections were prepared and routinely stained with hematoxylin-eosin.

### Flow cytometry

Flow cytometry was employed to characterize peripheral and CNS-infiltrating leukocytes in infected mice. Uninfected mice age-matched to 7 and 28 dpi were used as controls to prevent a potential age-related bias, especially in the infiltration of the CNS. As no differences were found (S2 Text), only uninfected mice age-matched to 7 dpi are shown in the main text. The blood samples were processed individually, but three mice were pooled to obtain adequate and robust amounts of leukocytes in the CNS samples.

First, the blood was collected from the right atrium of isoflurane-anesthetized mice and mixed with an equal volume of 10 mM EDTA in PBS on ice. Erythrocytes were lysed with ACK buffer [29] and the remaining cells were processed for flow cytometry. Following the blood collection, the mice were transcardially perfused with PBS, and the spinal cord, the brain stem, the cerebellum, and the left hemisphere were extracted and further processed on ice. The tissues were gently mechanically homogenized, filtered through a 70 μm cell strainer, and the leukocytes were separated from myelin using 30/70% Percoll (GE Healthcare; Chicago, Illinois, USA) gradient centrifugation [118]. Specifically, the cells were carefully collected from the 30/70% Percoll interphase and processed for flow cytometry.

After cell suspensions were prepared, anti-CD16/CD32 antibody (1:100; eBioscience; San Diego, California, USA; clone 93) was added for 10 minutes, being followed by a mixture of surface marker antibodies: APC-eFluor 780 anti-CD45 (1:100; eBioscience; clone 30F11); PE-Cy7 anti-CD11b (1:120; BioLegend; San Diego, California, USA; clone M1/70); FITC anti-F4/80 (1:100; BioLegend; clone BM8); PE anti-SiglecF (1:80; BioLegend; clone S17007L); APC anti-Ly6G (1:150; BioLegend; clone 1A8). The cells were stained for 30 minutes at 4°C in the dark, and Hoechst 33258 (1:22,500; Sigma Aldrich; St. Louis, Missouri, USA) was added 10 minutes before measurement to exclude dead cells. The samples were measured by CytoFLEX S (Beckman Coulter; Brea, California, USA; BC) using CytExpert (BC) and analyzed in FlowJo (v. 10.7.1). FMO controls were used for all markers; in the case of F4/80, isotype control was also included. The gating strategy for each tissue is shown in S2 Text. Cell populations of interest were characterized as follows: microglia: CD45^med^ CD11b^+^, macrophages/monocytes: CD45^+^ CD11b^+^ F4/80^+^ Ly6G^–^ SiglecF^med^, eosinophils: CD45^+^ CD11b^+^ F4/80^+^ Ly6G^–^ SiglecF^+^, neutrophils: CD45^+^ CD11b^+^ F4/80^+^ Ly6G^+^ SiglecF^low/med^, lymphoid cells: CD45^+^ CD11b^–^.

### Transcriptomic analysis

#### RNA isolation, library preparation, and sequencing

Total RNA was isolated from the whole dissected spinal cords of infected and age-matched control mice using TRIzol (Invitrogen). Four biological replicates per group were processed for each time point. Residual DNA was removed by TURBO DNA-free Kit (Invitrogen), and the quality and integrity of RNA were inspected by Bioanalyzer 2100 (Agilent; Santa Clara, California, USA). Pair-end (2x100 bp) sequencing was performed on the BGISEQ-500 platform.

#### Bioinformatic analysis

Raw reads were inspected for sequencing quality by FastQC (v. 0.11.5) [119], and low-quality nucleotides (Phred quality score <20) were removed using Trimmomatic (v. 0.39) [120]. Trimmed reads were mapped to transcriptome inferred from the reference genome (v. GRCm38) using RSEM (v. 1.3.3) [121], and transcripts with mapping rate >10 expected counts were considered as transcribed. Differential gene expression between the infected samples and the respective age-matched controls was calculated using DESeq2-package (v. 1.12.3) [122] in R (v. 3.5.2) at each time point. Transcripts with log_2_fold change (log_2_FC) >2 or <–2 and false discovery rate (FDR) of < = 0.05 were recorded as the differentially expressed. For a global overview of protein classes and metabolic pathways enriched at each time point, the reference transcriptome was translated using Transdecoder (v. 3.0.1) [123] and annotated by GhostKOALA (v. 2.2) [124] to KEGG database [125]. Upregulated (log_2_FC >2) and downregulated (log_2_FC <–2) transcripts with KEGG annotation were submitted to KEGG Mapper online tool to reconstruct metabolic pathways. Enriched metabolic pathways for each time point were identified by Fisher’s exact test (adjusted p-value <0.05) in R (v. 3.5.2).

### Laser capture microdissection and proteomic analysis

#### Embedding, cryosectioning, and laser microdissection

Cervical, thoracic, and lumbar spinal cord segments were extracted from mice previously perfused by heparinized (10 IU/ml) PBS and 4% PFA. The segments were post-fixed in 4% PFA for 5 hours at room temperature (RT), washed in PBS for 3×5 min, immersed in Tissue Freezing Medium (Leica; Wetzlar, Germany) for 30 min at RT, and frozen at –80°C. Ten-micrometer sections (CM3050 S Research Cryostat, Leica) were mounted onto membrane frame slides (MMI; Ecching, Germany) and stored at –80°C until use.

Laser microdissection was carried out using the MMI SmartCut system (Olympus CKX41 inverted microscope; Olympus SmartCut Plus software). Areas of interest were isolated from each spinal cord segment with a final volume of 7.5×10^6^ μm^3^ [126]. Specifically, the area of interest was the white matter of each spinal cord segment within up to 100 μm around the schistosomula. The randomly selected white matter of appropriate segments from uninfected mice was taken as a control sample. The microdissects were stored at –80°C until MS analysis. It was performed on either merged (cervical, thoracic, and lumbar all together, 7.5×10^6^ μm^3^ of each, n = 4, i.e., 22.5×10^6^ μm^3^ of microdissects per a replicate) or separated (n = 3, 7.5×10^6^ μm^3^ of microdissects per an individual segment replicate) spinal cord segments.

Additionally, the intestinal content of *T*. *regenti* schistosomula was also microdissected and analyzed. The intestinal content of schistosomula from 4 mice was pooled for one sample, and the experiment was performed in a triplicate. Due to a low yield of the microdissected material, only 1.3×10^6^ μm^3^ of the intestinal content was microdissected per sample.

#### MS analysis and data evaluation

Microdissects were processed for nanoflow liquid chromatography-MS analysis according to [126]. Briefly, proteins were denatured, reduced, and alkylated in one step using the In-StageTips method [127]. Raw data were processed and quantified with the MaxQuant software (v. 1.6.10.43) with a built-in Andromeda search engine against the protein databases of *M*. *musculus* (downloaded in June 2020 from www.uniprot.org) and *T*. *regenti* (trichobilharzia_regenti.PRJEB4662.WBPS14.protein) downloaded from the WormBase (parasite.wormbase.org) with parameter settings according to [126]. Quantification was performed using the label-free algorithm [128]. Further data analysis was done by Perseus software (v. 1.6.14.0) [129], where Student’s t-tests with permutation-based FDR calculation (250 randomizations) (FDR = 0.05, S0 = 0.1) were performed. Proteins considered to be reliably identifiable were further annotated and classified into protein groups according to the KEGG database [125].

### Immunohistochemistry

Ten-micrometer cryosections of spinal cords were prepared as already published [26] and stored at –80°C before use. After tempering, they were washed with PBS, permeabilized with 0.1% (v/v) Triton X-100 in PBS, blocked with 0.1% Triton X-100 1% (w/v) bovine serum albumin (BSA) in PBS, and incubated overnight at 4°C with primary antibodies: monoclonal rat anti-IL-4 (1:40; Abcam; Cambridge, UK; clone BVD4-1D11), monoclonal rat anti-IL-6 (1:200; Acris antibodies; Herford, Germany; clone MP5-20F3), monoclonal rat anti-MHC II (1:150; Abcam; clone NIMR-4), polyclonal rabbit anti-arginase-1 (Arg-1) (1:1000; Thermo Fisher Scientific; Waltham, Massachusetts, USA), polyclonal rabbit anti-glial fibrillary acidic protein (GFAP) (1:1000; Dako; Santa Clara, California, USA), polyclonal rabbit anti-ionized calcium-binding adaptor molecule 1 (Iba-1) (1:1000; Synaptic Systems; Göttingen, Germany). Negative rabbit serum was used as a control for Arg-1, GFAP, and Iba-1. The following day, the slides were washed with PBS, incubated with corresponding secondary antibodies (goat anti-rabbit F(ab’)2 AlexaFluor 594, goat anti-rabbit F(ab’)2 AlexaFluor 488, goat anti-rabbit Alexa Fluor 488 - all 1:1000; Cell Signaling Technology; Danvers, Massachusetts, USA; and goat anti-rat Alexa Fluor 568; 1:1000; Abcam) for 60 minutes at RT, washed with PBS and mounted in Fluoroshield with DAPI (Sigma Aldrich). Slides with detection of Arg-1 were captured by NIKON TiE 2 and processed in ImageJ (v. 1.8). Other slides were captured by Olympus BX51 equipped with Olympus DP-2 camera and processed in Quick Photo Micro (v. 3.0) and Gimp (v. 2.10.22). Per each time point, 4 mice were used, and at least 3 schistosomula-positive slides per mouse per antibody combination were analyzed. The mice used for this analysis were infected independently from other experiments presented herein.

### Light-sheet fluorescence microscopy

MHC II-EGFP C57BL/6J knock-in mice were perfused by ice-cold heparinized PBS and 4% PFA (pH 7.4), and their spinal cords were post-fixed in the PFA overnight at 4°C. The tissue was cleared in CUBIC-1 solution [25% (w/v) urea, 25% (w/v) Quadrol, 15% (v/v) Triton X-100 (all Sigma-Aldrich) in dH_2_O] for at least 5 days (or until the tissue was transparent) in the dark, orbital shaking at 37°C. Afterwards, the tissue was washed [0.5% (w/v) BSA, 0.01% (w/v) sodium azide, 0.1% (v/v) Triton X-100 in PBS)] in the dark, orbital shaking at 37°C for 5 hours. Washed samples were stained with DRAQ5 (Thermo Fisher Scientific) (1:7500) for 2 days in the dark, rocking at 4°C. Finally, the tissue was incubated in CUBIC-2 solution [22.5% (w/v) urea, 9% (v/v) triethanolamine (Sigma-Aldrich), 45% (w/v) sucrose, 0.1% (v/v) Triton X-100 in dH_2_O; refractive index = 1.47)] at least for 5 days in the dark, orbital shaking at 37°C. The tissue clearing protocol was adapted from [130] and modified by Jaromír Novák and Jan Pačes (Department of Cell Biology, Faculty of Science, Charles University). Samples were mounted in 1% (w/v) low-melt agarose and processed on Zeiss Lightsheet Z.1 using 10× illumination and 20× detection objectives. Data were analyzed and processed in ZEN (black edition) (v. 9.2.8.54; Zeiss), Arivis (v. 3.1.3; Arivis), and DaVinci Resolve (v. 16.2.7.010; Blackmagic design). The mice used for this analysis were infected independently from other experiments presented herein.

### Evaluation of the blood-brain barrier integrity

The integrity of the blood-brain barrier was evaluated by the extravasation of Evans blue into the nervous tissue [131]. The mice were subtly anesthetized (100 mg/kg ketamine, 10 mg/kg xylazine), and 200 μl of 2% (w/v) Evans blue were injected into a lateral tail vein. After 1 h, the mouse was transcardially perfused by 40 ml of heparinized (10 IU/ml) PBS and the spinal cord, brain stem, cerebellum, and hemispheres were isolated and weighed. The tissue was then mechanically homogenized in 50% of trichloroacetic acid (spinal cords, brain stems, and cerebella in 100 μl, hemispheres in 300 μl), centrifuged (20 min, 10,000×g) and the supernatant was diluted 4-fold with ethanol. The fluorescence intensity was measured at 620/680 nm and converted to the amount of Evans blue using a standard curve. The mice used for this analysis were infected independently from other experiments presented herein.

### Apoptosis assays

DNA fragmentation, a hallmark of apoptotic cells, was detected on cryosections of the spinal cords (see “Immunohistochemistry” for preparation) by Click-iT Plus TUNEL Assay with Alexa Fluor 488 dye (Thermo Fisher Scientific) according to the manufacturer’s instructions. Then the slides were mounted in VectaShield with DAPI to stain all nuclei and examined by a fluorescence microscope (Olympus BX51). The images were processed in ImageJ, by which the proportion of TUNEL+ nuclei around and within the schistosomula was calculated and compared to uninfected mice. Per each time point, 4 mice were used, and at least 2 schistosomula-positive slides per mouse were analyzed. The mice used for this analysis were infected independently from other experiments presented herein.

Apoptotic effects of *T*. *regenti* antigens were tested *in vitro* on Neuro2a cell line and primary mixed glial cultures. Neuro2a were obtained from Ondřej Honc (Department of Physiology, Faculty of Science, Charles University) and grown in Dulbecco’s modified Eagle’s medium supplemented by 10% (v/v) fetal bovine serum and 1% (v/v) penicillin/streptomycin (all from Lonza; Basel, Switzerland). Mixed glial cultures were prepared from newborn mouse pups and grown as already described [25]. The cells were seeded into 12-well plates and treated with a soluble fraction of schistosomula homogenate (5 and 50 μg/ml; see [25] for a detailed preparation) for 48 hours. Staurosporine (final concentration 0.1 μM) was used as a positive control. Next, the cells were washed by PBS, trypsinized, and stained by Dead Cell Apoptosis Kit with annexin V-Alexa Fluor 488 and propidium iodide (Thermo Fisher Scientific) according to the manufacturer’s instructions. The samples were measured by LSR II flow cytometer using Diva (Becton Dickinson; Franklin Lakes, New Jersey, USA) and analyzed in FlowJo (v. 10.7.1).

### Statistical analyses

Statistical analyses and data visualization were conducted in GraphPad Prism (v. 9) if not already stated otherwise. Particular tests applied are indicated in the figure legends, p-values <0.05 were considered significant and are indicated as follows: *p<0.05, **p<0.01, ***p<0.001. The exact p-value is also shown if it was 0.05–0.10. Individual data are presented within the graphs, or “n” is clearly stated if summary statistics (mean ± standard deviation) were used for better graphical representation.

## Supporting information

S1 Table Spinal cord transcriptomic data. (XLSX)

S2 Table Microdissection proteomic data. (XLSX)

S1 Text Detailed description of behavioral tests. (DOCX)

S2 Text Gating strategies and flow cytometry data not presented in the main text. (DOCX)

S1 Fig Behavioral data not presented in the main text. (TIF)

S2 Fig Evaluation of the blood-brain barrier integrity by Evans blue in uninfected (0 dpi) and infected (7 dpi) mice. **(A**) Representative images of extracted spinal cords and brains. **(B)** Quantification of Evans blue in the nervous tissue. Data were evaluated by unpaired t-test, p values are shown.
(TIF)

S1 Video Beam walking, uninfected and infected (7 dpi) mice. (MP4)

S2 Video Light-sheet fluorescence microscopy, the uninfected mouse (0 dpi). (MP4)

S3 Video Light-sheet fluorescence microscopy, the infected mouse (7 dpi). (MP4)

S4 Video Light-sheet fluorescence microscopy, the infected mouse (14 dpi). (MP4)

S5 Video Visualization of the virtual box used for quantification of MHC II+ volume around schistosomula. (MP4)
