# Supplementary material for: Mechanisms of the host immune response and helminth-induced pathology during Trichobilharzia regenti (Schistosomatidae) neuroinvasion in mice
Source: PLoS Pathog. 2022 Feb 4;18(2):e1010302. doi: 10.1371/journal.ppat.1010302 (PMC8849443; doi:10.1371/journal.ppat.1010302)
Supplement: S1 Text — (DOCX) [file ppat.1010302.s003.docx]

**S1 Text: Detailed description of behavioral tests**

**General notes**

All behavioral testing was done during the day (between 09:00 am and 06:00 pm). The mice were single-housed with free access to food and water. Silent relaxation music was used as a noise background prior to and during the testing to minimize the effects of eventual uncontrolled sounds from outside.

Most of the behavioral tests (elevated plus maze; open field; marble burying; novelty-induced hypophagia; Y-maze; grid test) were recorded using a digital video camera (Logitech C920) placed directly overhead and analyzed offline. Evaluation of total distance walked, or immobility was done in EthoVision 14 (Noldus, Netherlands), while specific behaviors were marked using BORIS [1].

**Elevated plus maze test**

The elevated plus maze is a behavioral setup for anxiety testing in rodents, based on a conflict between avoidance of exposed areas and motivation to explore [2,3]. The cross-shaped maze has two closed arms, enclosed by opaque walls, and two open arms without such walls, and is elevated above the floor. Mice prefer protected shelters over well-lit and elevated areas, and the tendency to avoid the open arms is taken as a measure of anxiety.

Our maze was made from light grey plastic and had four arms, 30×5 cm each, and was elevated 65 cm above the floor. The walls of the closed arms were 15 cm high, and the illumination varied between 20-36 lux in different parts of the maze. A mouse was always placed into the middle of the maze, facing an open arm, and left undisturbed for 5 min.

**Total distance** walked and the total **number of arm entrances** were evaluated as a measure of general activity. **Time spent in the open arms**, **risk assessment** behavior, and **looking down** from the maze were scored as a measure of anxiety. Arm entrance was counted whenever the mouse placed all four limbs into the arm. Risk assessment was counted when the mouse poked its head out of a closed arm while the body or at least hindlimbs remained inside.

**Open field test**

The open field test is commonly used to assess spontaneous locomotion and anxiety [3–7].

In our experiment, a mouse was placed into the center of a brightly illuminated (220 lux) white chipboard square arena (50×50 cm surrounded by 40-cm walls) and left undisturbed for 10 min. **Total distance walked** during the session was used to evaluate locomotor activity. **Time spent in the center** of the arena was used as a measure of anxiety, as rodents tend to spend more time in corners or near the walls.

**Beam walking test**

The beam walking test assesses locomotor abilities and sensorimotor coordination [4,5,7]. We used a 1-m long wooden beam elevated 90 cm above the floor. One end of the beam was closed by a barrier, while the other was placed on an open home cage. The animal was placed on the blocked end of the beam, facing the homecage. Its goal was to traverse the beam and reach the home cage. The behavior of the mouse was watched by two observers, one on each side of the beam. In case of a fall, the mouse was gently caught by the experimenter to prevent injuries.

The mice were first trained on a 10 mm wide beam. To familiarize with the task, they were released from 1/4 and then twice from 1/2 of the beam length before two full-length trials. This was followed by training on a narrow (5 mm) beam, with two half-length trials and two full-length trials. Only the full-length trials were evaluated.

**Time to traverse the beam** was measured using a stopwatch, from the release to the moment when the mouse's nose reached the edge of the cage. If the mouse did not reach the homecage before 60 seconds or fell from the beam, it was counted as the maximum time (60 s).

Footslips, near-falls, and falls were counted. Other gait abnormalities, such as belly dragging, were also noted. **Error score** was calculated as the total number of footslips (maximum 20), with near-fall scoring as 5 slips and a fall or belly dragging scoring as 20 slips.

**Bar holding test**

The bar holding test [6] is a task focused on forelimb grasping strength and endurance. A 2-mm thick cotton rope was stretched tight, approximately 50 cm above the floor of the open field arena. The floor of the arena was cushioned to prevent injuries. The tested mouse was made to grasp the rope with its forepaws and was left hanging from it for 60 seconds. **Time to jump/fall** from the rope was measured using a stopwatch. The test was repeated twice, and the better score was counted.

**Marble burying test**

The marble burying test [8] is based on the tendency of laboratory rodents to bury foreign objects under the bedding, which is generally taken as a sign of anxious or compulsive behavior.

We performed the test in a transparent cage 20.5×26.5×14 cm with a wire lid, and the illumination level was 30 lux. The cage was filled with a 5-cm layer of fresh wood chips. Twelve transparent glass marbles (16 mm diameter) were arranged into a regular grid in one half of the cage; the other half of the bedding was left empty.

Each mouse was placed in the empty half of the cage and left undisturbed for 30 minutes. **The number of marbles buried** to at least 2/3 of their size was counted.

**Novelty-induced hypophagia = Novelty-suppressed feeding**

Rodents are generally reluctant to eat novel food, and this behavior is used as a proxy of anxiety [3]. Our setting consisted of an opaque box (28×43.7×16 cm; illumination: 160 lux) without bedding, covered with a wire lid. In the middle, a small plastic Petri dish with novel food was placed. The bait consisted of three sugar-flavored oat flakes (soaked with a 30% sucrose solution and dried), unfamiliar to the mice and different from the standard chow in both appearance and taste. The tested mouse was placed into a corner of the box and left undisturbed for 30 min. **The amount of oat flakes eaten** was noted (the flakes were weighed before and after the session), and **the latency to first tasting** the food was noted.

**Spontaneous alternation in the Y-maze**

In the Y-maze test [9], we used a three-armed maze (arm size 35×6 cm, arms marked ‘A’, ‘B’ and ‘C’) with 20-cm high walls, made of light grey plastic and dimly illuminated (5 lux). Each mouse was placed at the end of the arm ‘A’ facing the center and left to explore the maze for 8 min. From the video recordings, the order of visits of individual arms was noted. Any three subsequent arm visits made a triad, which was counted either as correct, if all the three arms were visited, or incorrect, if the mouse failed to visit one of the three, or visited one arm repeatedly. Arm entrance was counted when all four limbs of the mouse were inside. **The proportion of correct triads** to all triads was used as a measure of spontaneous alternation, which is dependent on spatial working memory. **Total distance walked** and the **total number of arm entrances** were used as a measure of the general activity of the animal.

**Grid test**

In the grid test of motor abilities and endurance [7,10,11], the tested mouse was placed onto a wire mesh (mesh size 6×6 mm, wire diameter 0.5 mm) stretched in a 50×50 cm wooden frame. The frame was then turned upside-down over 2 seconds, with the mouse’s head descending first and placed onto the open field arena. **The latency to fall or jump** from the grid was measured using a stopwatch; the maximum duration of a trial was 5 min. The test was repeated three times, and only the best score was counted.

**Tail suspension test**

Tail suspension test was used for assessment of depression-like behavior or behavioral despair [7,12]. Each mouse was suspended by the tail (using a sticky tape holding the last 2 cm of the mouse’s tail) and left hanging for 5 min. The mice could react either by struggling or by despair (motionless hanging), which was recorded by a camera from aside. **Immobility** **duration** was detected by the EthoVision software (Activity state – immobile).

**Forced swimming**

The forced swimming task is another test of behavioral despair [12]. The tested mouse was placed into a large graduated cylinder (7.5 cm diameter) filled with water (25 °C) so that the mouse could reach neither the bottom nor the edges of the cylinder. The cylinder was video recorded from aside. After 5 min, the trial was terminated, and the mouse was rescued from the water using a pasta ladle. **Immobility** **duration** was detected by the EthoVision software (Activity state – immobile).

**Footprint analysis**

In this simple method of gait analysis [11], the paws of a mouse were covered with non-toxic finger paint (red color for the forepaws and blue for the hindpaws), and the mouse was released into a corridor (10×50 cm, walls 30 cm high) with a floor covered by white filter paper. **Step length** and **step width** of both fore- and hindlimbs were analyzed.

**References**

1. Friard O, Gamba M. BORIS: a free, versatile open‐source event‐logging software for video/audio coding and live observations. Fitzjohn R, editor. Methods Ecol Evol. 2016;7: 1325–1330. doi:10.1111/2041-210X.12584

2. Komada M, Takao K, Miyakawa T. Elevated plus maze for mice. J Vis Exp. 2008; 1088. doi:10.3791/1088

3. Riebe CJ, Wotjak CT. A practical guide to evaluating anxiety-related behavior in rodents. In: Szallasi A, Bíró T, editors. TRP Channels in Drug Discovery. 2012. pp. 167–185. doi:10.1007/978-1-62703-095-3_10

4. Curzon P, Zhang M, Radek RJ, Fox GB. The behavioral assessment of sensorimotor processes in the mouse: Acoustic startle, sensory gating, locomotor activity, rotarod, and beam walking. 2nd ed. Methods of Behavior Analysis in Neuroscience. 2nd ed. 2009.

5. Hölter SM, Glasl L. High-throughput mouse phenotyping. In: Lane E, Dunnett S, editors. Animal Models of Movement Disorders. 2011. pp. 109–133. doi:10.1007/978-1-61779-298-4_7

6. Justice JN, Carter CS, Beck HJ, Gioscia-Ryan RA, McQueen M, Enoka RM, et al. Battery of behavioral tests in mice that models age-associated changes in human motor function. Age (Omaha). 2014;36: 583–595. doi:10.1007/s11357-013-9589-9

7. Mann A, Chesselet MF. Techniques for motor assessment in rodents. 2nd ed. In: LeDoux M, editor. Movement Disorders. 2nd ed. 2014. pp. 139–157. doi:10.1016/C2012-0-00370-5

8. Deacon RM. Digging and marble burying in mice: simple methods for in vivo identification of biological impacts. Nat Protoc. 2006;1: 122–124. doi:10.1038/nprot.2006.20

9. Kraeuter A-K, Guest PC, Sarnyai Z. The Y-maze for assessment of spatial working and reference memory in mice. Pre-Clinical Models. 2019. pp. 105–111. doi:10.1007/978-1-4939-8994-2_10

10. Deacon RM. Measuring the strength of mice. J Vis Exp. 2013; 2610. doi:10.3791/2610

11. Sugimoto H, Kawakami K. Low-cost protocol of footprint analysis and hanging box test for mice applied the chronic restraint stress. J Vis Exp. 2019; 59027. doi:10.3791/59027

12. Porsolt RD, Brossard G, Hautbois C, Roux S. Rodent models of depression: Forced swimming and tail suspension behavioral despair tests in rats and mice. Curr Protoc Neurosci. 2001;14: Unit 8.10A. doi:10.1002/0471142301.ns0810as14
