# Supplementary material for: Mechanisms of the host immune response and helminth-induced pathology during Trichobilharzia regenti (Schistosomatidae) neuroinvasion in mice
Source: PLoS Pathog. 2022 Feb 4;18(2):e1010302. doi: 10.1371/journal.ppat.1010302 (PMC8849443; doi:10.1371/journal.ppat.1010302)

**S2 Text: Gating strategies and flow cytometry data not presented in the main text**

**The representative gating strategy in the spinal cord**


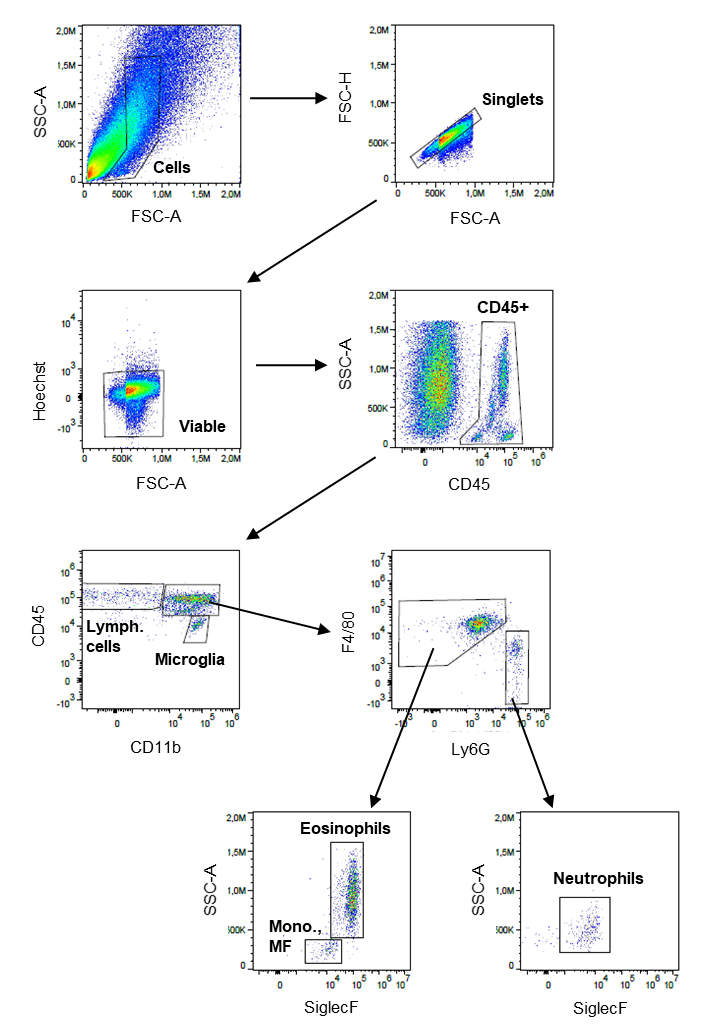


**The representative gating strategy in the brain stem**


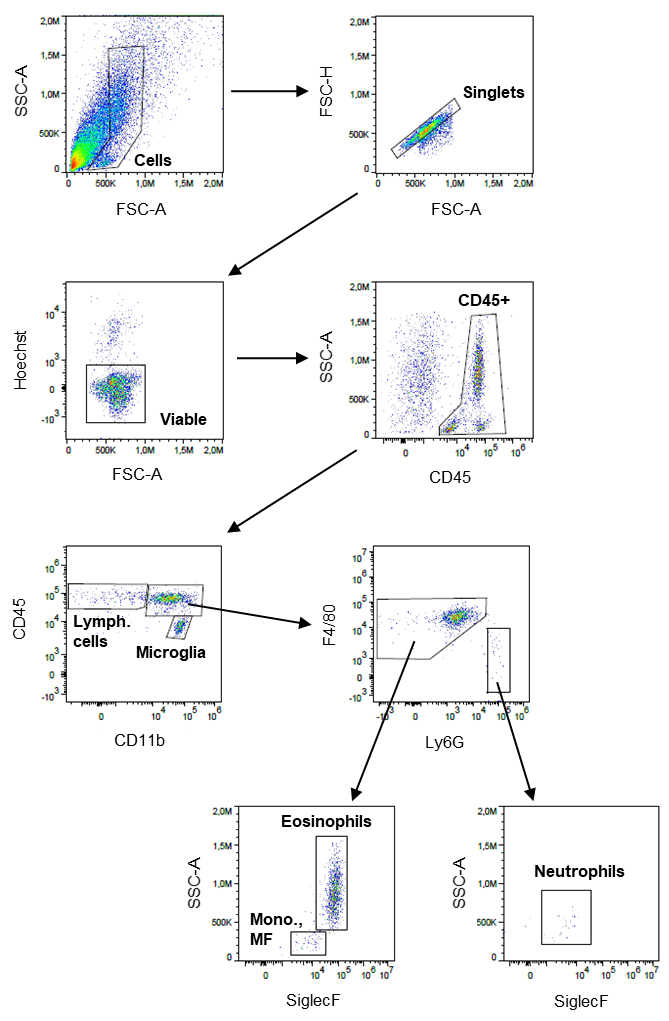


**The representative gating strategy in the cerebellum**


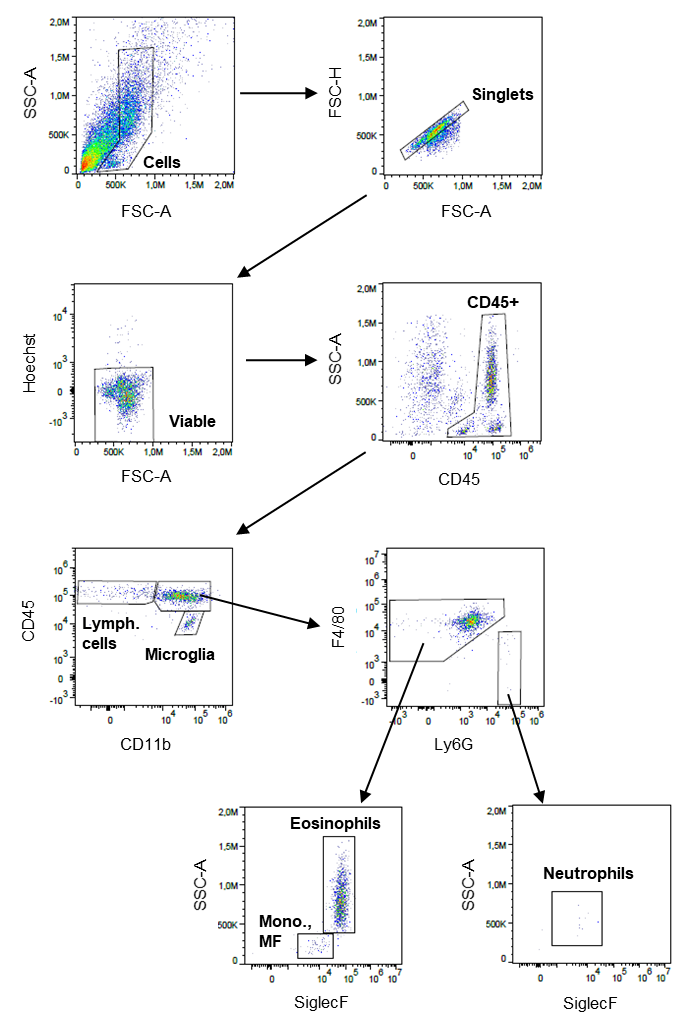


**The representative gating strategy in the hemisphere, fluorescence minus one (FMO) or isotypes controls are also depicted**


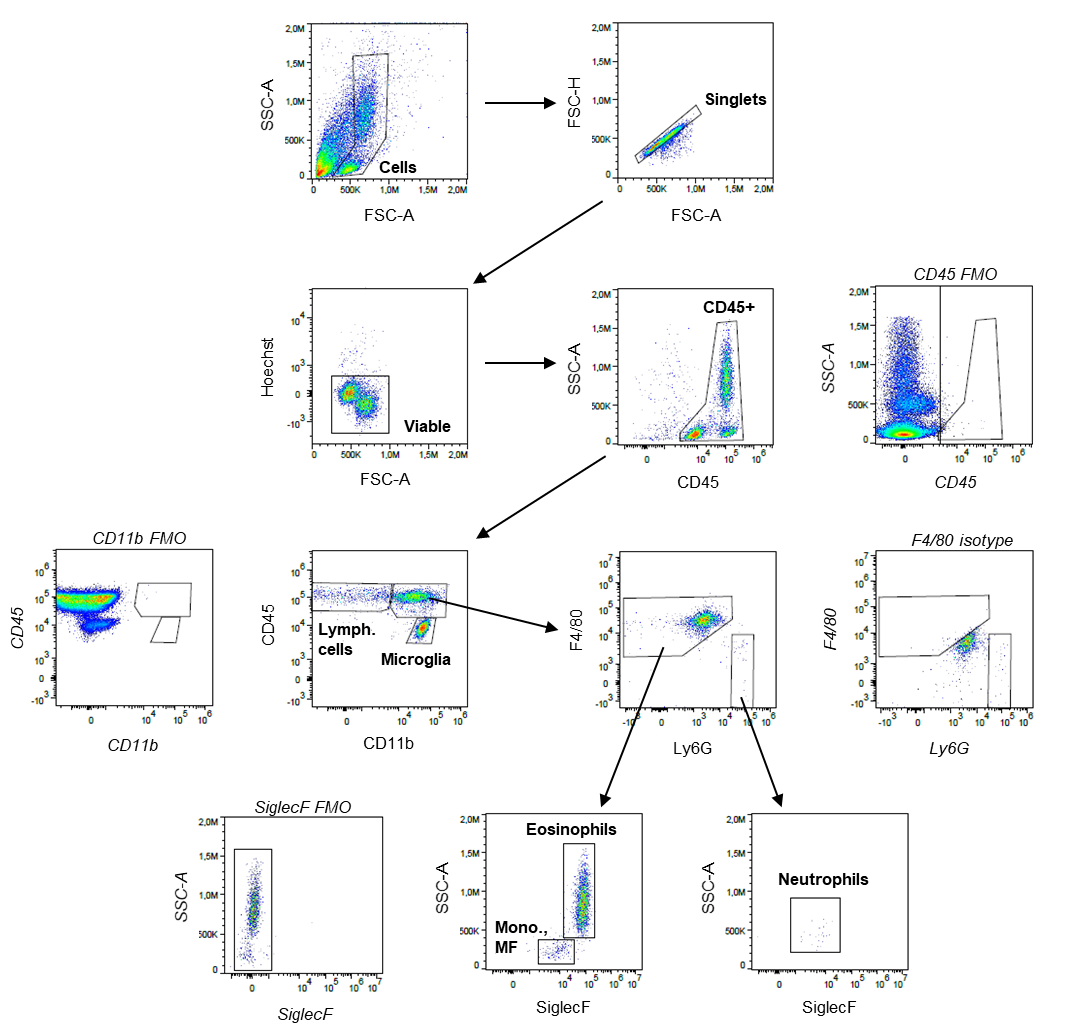


**The comparison of immune cells in the CNS of 9- and 12-week old naïve mice (age-matched to infected mice 7 and 28 dpi)**


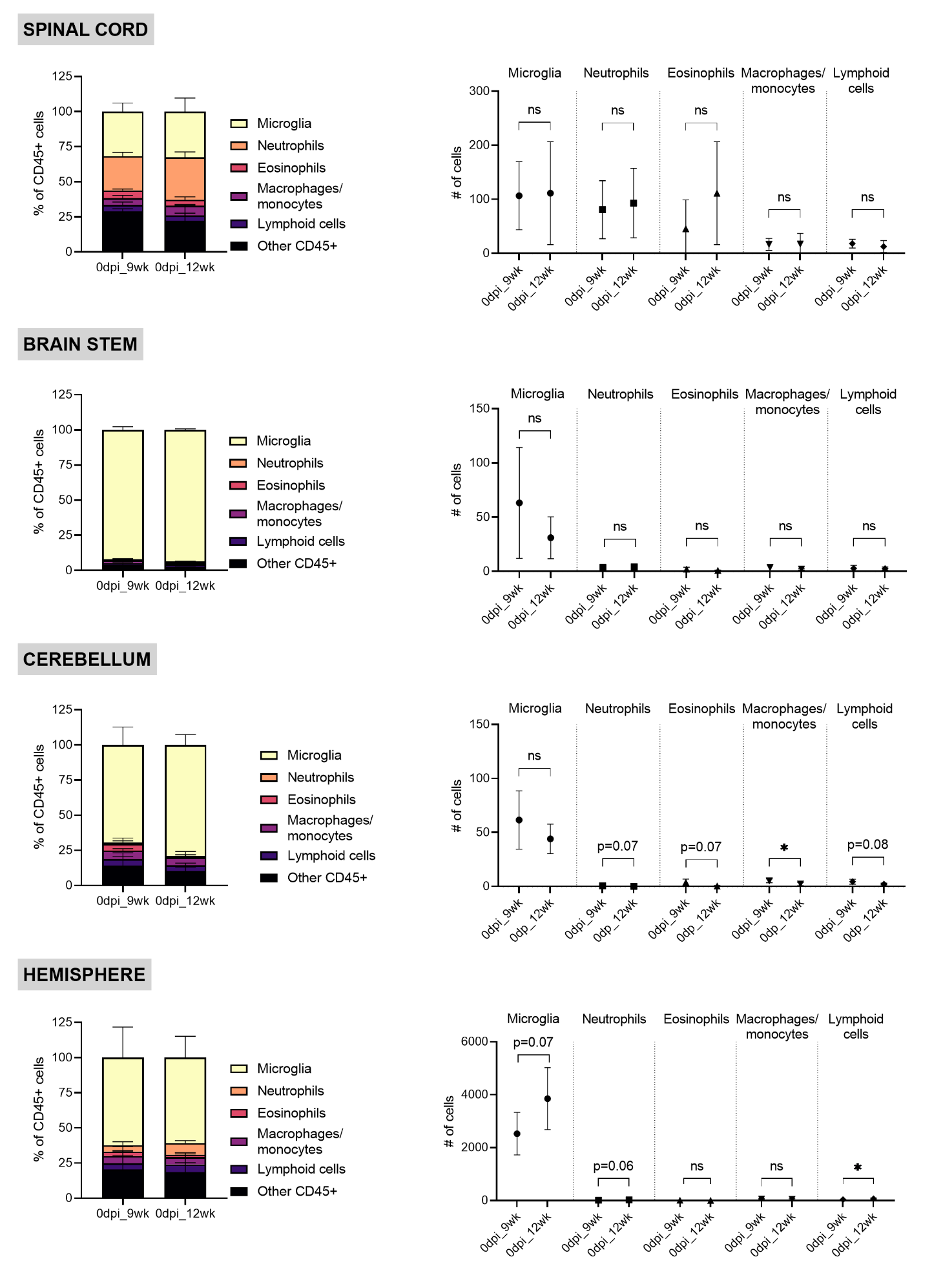


**The representative gating strategy in the blood**


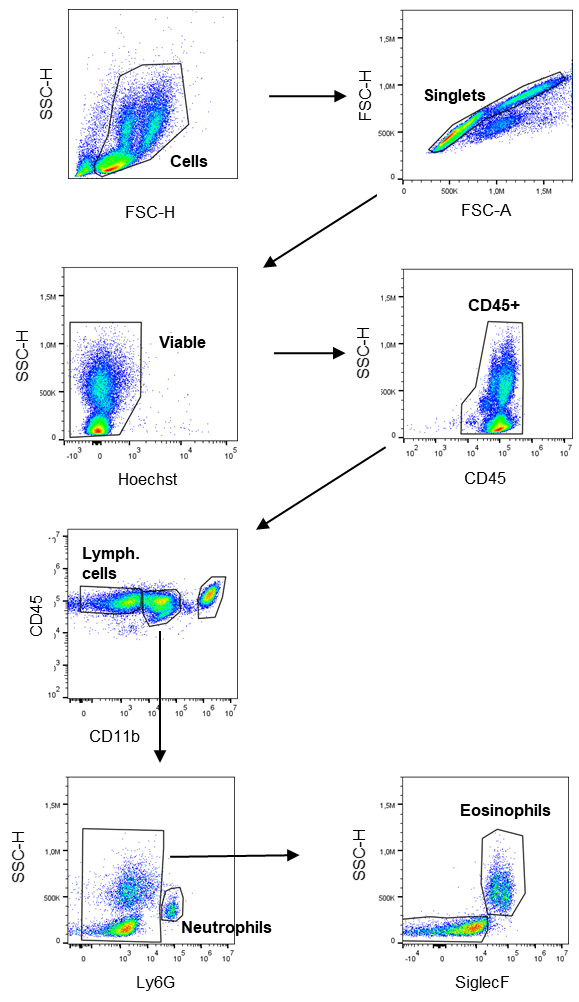

Supplement: S2 Text — (DOCX) [file ppat.1010302.s004.docx]
