# Supplementary figures and images for: Mechanisms of the host immune response and helminth-induced pathology during Trichobilharzia regenti (Schistosomatidae) neuroinvasion in mice
Source: PLoS Pathog. 2022 Feb 4;18(2):e1010302. doi: 10.1371/journal.ppat.1010302 (PMC8849443; doi:10.1371/journal.ppat.1010302)

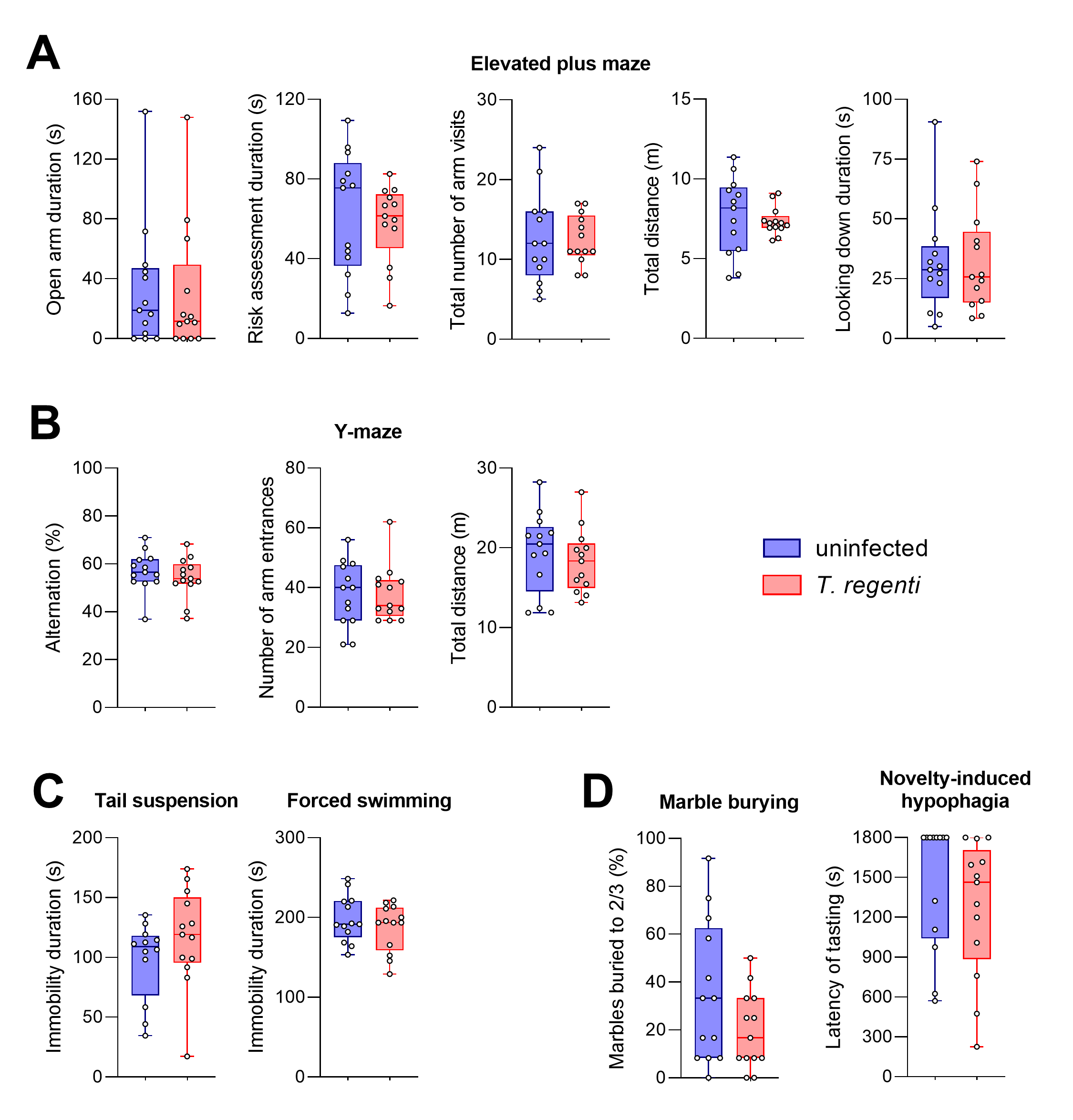

Supplement: S1 Fig — (TIF) [file ppat.1010302.s005.tif]

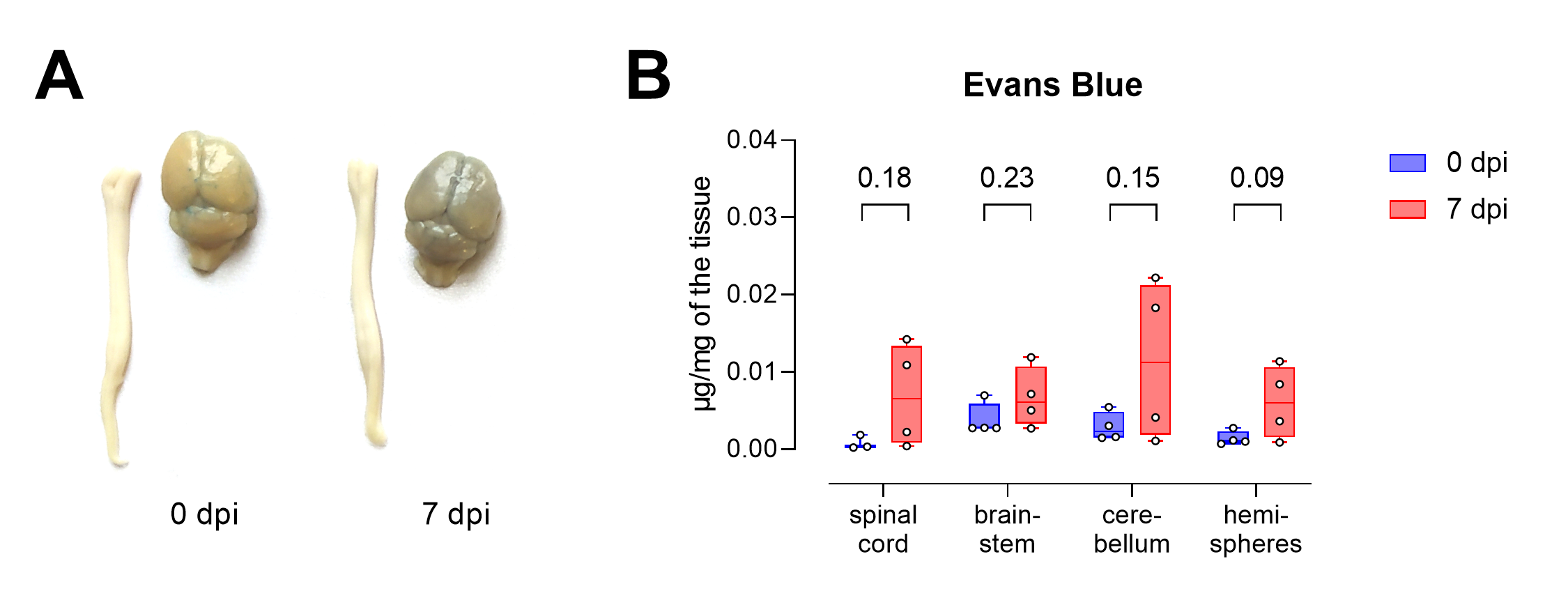

Supplement: S2 Fig — (A) Representative images of extracted spinal cords and brains. (B) Quantification of Evans blue in the nervous tissue. Data were evaluated by unpaired t-test, p values are shown. (TIF) [file ppat.1010302.s006.tif]
